# Exhausted intratumoral Vδ2^−^ γδ T cells in human kidney cancer retain effector function

**DOI:** 10.1038/s41590-023-01448-7

**Published:** 2023-03-16

**Authors:** Chiara Rancan, Marcel Arias-Badia, Pranay Dogra, Brandon Chen, Dvir Aran, Hai Yang, Diamond Luong, Arielle Ilano, Jacky Li, Hewitt Chang, Serena S. Kwek, Li Zhang, Lewis L. Lanier, Maxwell V. Meng, Donna L. Farber, Lawrence Fong

**Affiliations:** 1 Division of Hematology/Oncology, Department of Medicine, University of California, San Francisco, CA USA; 2 Department of Microbiology and Immunology, Columbia University Medical Center, New York, NY USA; 3 The Taub Faculty of Computer Science and Faculty of Biology, Technion-Israel Institute of Technology, Haifa, Israel; 4 Helen Diller Family Comprehensive Cancer Center, University of California, San Francisco, CA USA; 5 Parker Institute for Cancer Immunotherapy, University of California, San Francisco, CA USA; 6 Department of Microbiology and Immunology, University of California, San Francisco, CA USA; 7 Department of Urology, University of California, San Francisco, CA USA

**Keywords:** Immunosurveillance, Tumour immunology, Gammadelta T cells

## Abstract

Gamma delta (γδ) T cells reside within human tissues including tumors, but their function in mediating antitumor responses to immune checkpoint inhibition is unknown. Here we show that kidney cancers are infiltrated by Vδ2^−^ γδ T cells, with equivalent representation of Vδ1^+^ and Vδ1^−^ cells, that are distinct from γδ T cells found in normal human tissues. These tumor-resident Vδ2^−^ T cells can express the transcriptional program of exhausted αβ CD8^+^ T cells as well as canonical markers of terminal T-cell exhaustion including PD-1, TIGIT and TIM-3. Although Vδ2^−^ γδ T cells have reduced IL-2 production, they retain expression of cytolytic effector molecules and co-stimulatory receptors such as 4-1BB. Exhausted Vδ2^−^ γδ T cells are composed of three distinct populations that lack *TCF7*, are clonally expanded and express cytotoxic molecules and multiple Vδ2^−^ T-cell receptors. Human tumor-derived Vδ2^−^ γδ T cells maintain cytotoxic function and pro-inflammatory cytokine secretion in vitro. The transcriptional program of Vδ2^−^ T cells in pretreatment tumor biopsies was used to predict subsequent clinical responses to PD-1 blockade in patients with cancer. Thus, Vδ2^−^ γδ T cells within the tumor microenvironment can contribute to antitumor efficacy.

## Abstract (web summary)

γδ T cells contribute to cancer immunity by killing tumor cells, but their function in the context of immune checkpoint inhibition is less clear. Here the authors show that a Vδ2^−^ subset of γδ T cells in human kidney tumors phenotypically resembles exhausted T cells yet retains this cytolytic function and can be used to predict response to immune checkpoint inhibition.

## Main

Immune checkpoint inhibition (ICI) has brought remarkable advances in the treatment of many cancer types. Antibodies targeting immune checkpoint receptors or their ligands, such as the inhibitory receptors cytotoxic T-lymphocyte-associated antigen-4 (CTLA-4), programmed death-1 (PD-1) and its ligand PD-L1, can induce clinical responses in a range of cancers^1^. ICIs are now the first-line therapies to treat patients with metastatic renal cell carcinoma (mRCC). Metastatic RCCs account for 20–25% of all patients with RCC and with current therapies show poor 5 year survival prognosis. PD-1 blockade in combination with anti-CTLA-4 or angiogenesis inhibitors leads to improved overall response and overall survival in randomized trials^2–4^. Additionally, in the IMmotion150 phase 2 trial, anti-PD-L1 agent (atezolizumab) in combination with anti-VEGF (bevacizumab) was associated with improved overall response rates^5^. Despite these promising results in mRCC, a substantial proportion of patients do not benefit from treatment with ICI. Response to therapy depends on the level of expression of the targeted checkpoint and the extent of immune cell infiltrate, among other stratifying parameters^6,7^, but there are no established biomarkers that are routinely used to select treatment in mRCC. Even though most research efforts so far have focused on how these therapies reinvigorate conventional αβ T cells, promising data showing antitumor activity by γδ T cells in RCC^8–13^ might suggest that new strategies aiming at effective infiltration or reinvigoration of cytotoxic γδ T cells into the tumor could be beneficial.

γδ T cells are one such effector population with unknown roles in ICI. They differ from αβ T cells on the basis of the composition of their T-cell receptors (TCRs), which consist of a gamma chain paired to a delta chain instead of an alpha/beta heterodimer. Unlike αβ T cells, γδ T cells exert antigen-driven cytotoxicity against target cells in an MHC-independent fashion and can recognize a broad variety of antigens^14,15^. Upon target recognition, γδ T cells become rapidly activated and induce clonotypic responses, allowing for fast-acting immunity^14^. Subsets of γδ T cells are further classified according to the expression of different TCR chains. Vγ9Vδ2^+^ (referred to as Vδ2^+^ onwards) γδ T cells are the predominant population in the periphery, accounting for about 5–10% of total circulating lymphocytes and 70–90% of circulating γδ T cells. Vδ2^+^ cells indirectly recognize phosphoantigens, small nonpeptidic molecules produced by bacteria and tumor cells, through a mechanism positively and negatively regulated by endogenous butyrophilin proteins^16^, which may have a direct role in successfully coordinated αβ and γδ T-cell antitumor responses in ovarian cancer^17^. The other γδ T cells, collectively grouped under the Vδ2^−^ cell umbrella, are more abundant in tissues and have been shown to recognize a broad variety of antigens, ranging from stress-upregulated self-molecules, such as MHC I Chain-related proteins to lipid-loaded CD1 molecules^18^. Overall, γδ T cells can play multiple and sometimes opposing functions, especially in the tumor context. Both Vδ2^+^ and Vδ2^−^ cells have been shown to (1) mediate potent antitumor activity through IFNγ, TNF and cytotoxic granules^19,20^, (2) perform both pro-inflammatory and immunosuppressive functions^21–24^, and (3) in the case of Vδ2^+^ cells, perform antigen uptake and presentation to other T cells^25,26^. Perhaps reflecting their functional diversity, the frequency of tumor-infiltrating γδ T cells has been associated with both favorable and unfavorable clinical outcomes^27,28^. Like αβ T cells, γδ T cells in tumors can also express immune checkpoints^29–34^, but their effect in cell function is still unclear. Interestingly, most reports identified Vδ2^−^ T cells as the PD-1-expressing γδ T-cell subset. Given the heterogeneity and the functional plasticity of γδ T cells, we speculate that these cells may respond differently than αβ T cells to ICI.

In this Article, using high-dimensional single-cell approaches, we show the composition and function of intratumoral γδ T cells in samples from patients with RCC and from healthy donors. We identified a nonclonal subset of Vδ2^−^ T cells expressing markers of terminal exhaustion, including PD-1, TIGIT and TIM-3. These cells proved to be enriched in tumors, but nearly absent in healthy tissues. This population maintained expression of effector cytokines and perforin at similar levels to nonexhausted cells and were able to kill autologous tumor cells in ex vivo co-cultures. The presence of a molecular signature derived from the Vδ2^−^ subset correlated with improved clinical outcomes in patients with RCC and urothelial cancer who were treated with ICI. In summary, our data identify unique spatial and functional features of γδ T cells that could be exploited for immunotherapy of tumors.

## Results

### Pan-γδ analysis shows tumor Vδ2^−^ cells expressing PD-1 and 4-1BB

**Fig. 1:**
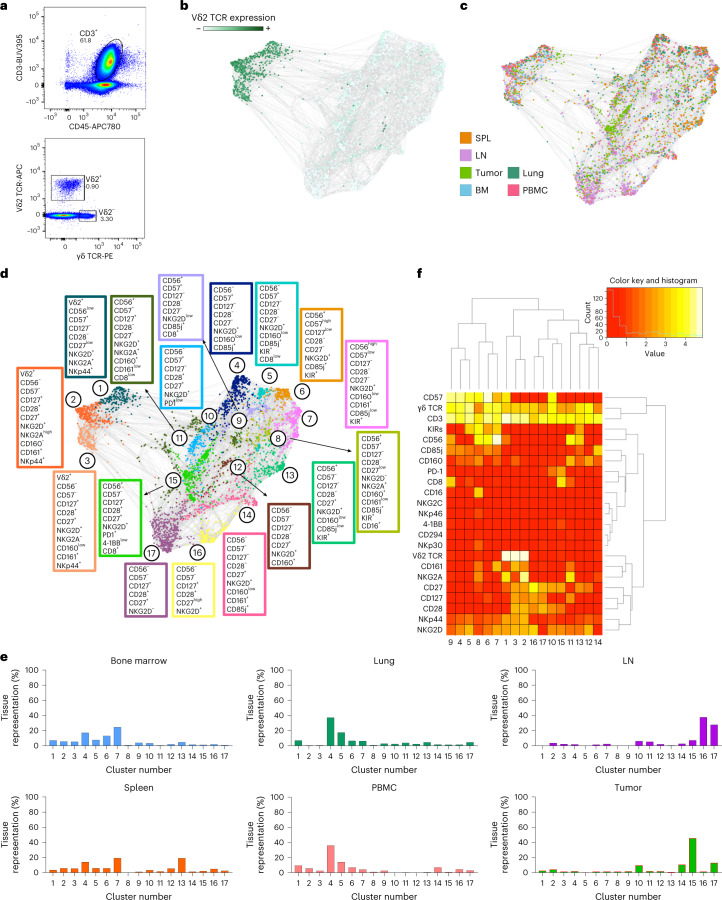
Tumors are enriched with Vδ2^−^ γδ T cell populations. **a**–**f**, γδ T cells from six RCC tumors and tissues from five healthy donors (eight LN, five spleens (SPL) and bone marrows (BM), and four lungs and PBMC samples) were analyzed by high-parameter flow cytometry. Data from each sample were gated on γδ TCR^+^ cells and compiled together in a force-directed graph. **a**, Representative flow cytometry dot plots showing the gating strategy for Vδ2^−^ and Vδ2^+^ T cells in RCC samples. **b**, Force-directed graph highlighting Vδ2 TCR expression is shown. **c**, Force-directed graph colored by tissue source is shown. **d**, Force-directed graphs of γδ T cells are shown. Phenotypically similar nodes (clusters) are highlighted on force-directed graphs with the same color. Each color corresponds to a phenotypically distinct cluster (17 clusters total). Enriched markers for each cluster are highlighted in color-matched text boxes. **e**, Bar plots showing per tissue total cell frequencies of each phenotypic cluster. **f**, Heat map showing the differential expression of surface markers used for clustering. Dendrograms show the results of hierarchical clustering used to group similar clusters (columns) and markers with similar cluster distributions (rows).

**Extended Data Fig. 1:**
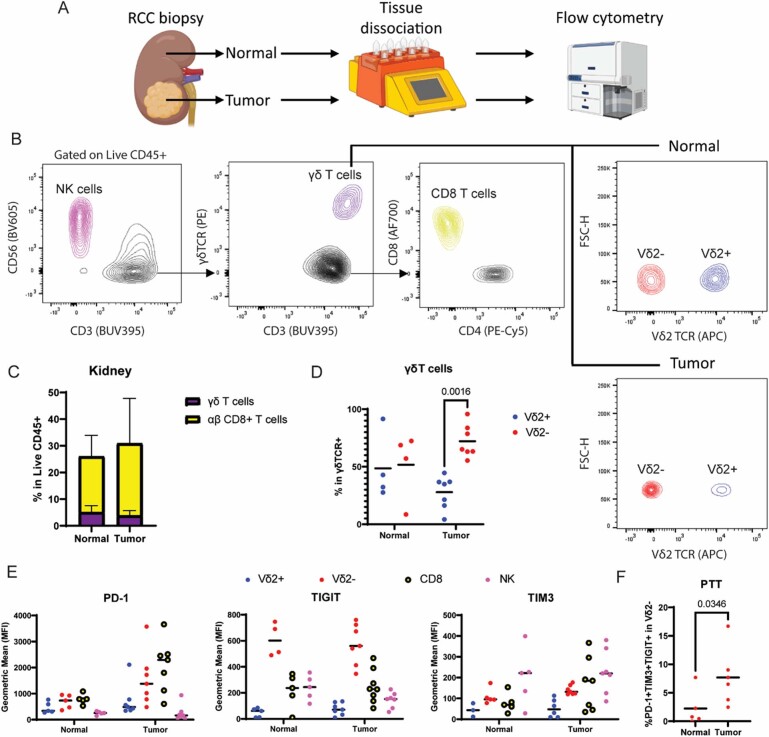
Characterization of αβ and γδ T cells in normal and RCC tumor samples. **a**. Schematic diagram showing the workflow performed on healthy kidney and RCC tumor samples. **b**. Gating strategy in flow cytometry analysis of 5 healthy tissue and 7 RCC samples (left). Contour plots showing expression of lineage markers CD56 (NK), CD3 and γδTCR (T cells), CD8 vs CD4 (αβ T cells), and Vδ2 TCR in normal vs tumor. **c**. Stacked bar plot showing cell frequencies of αβ and γδ T cells in normal and RCC samples. No significance found by one-sided Mann-Whitney test for each cell type. Error bars represent standard deviation. **d**. Percentage of Vδ2+ and Vδ2- subsets within overall γδ T cells in normal and tumor samples. Each dot represents one sample. Significance assessed by 2-way ANOVA multiple comparison test. **e**. Expression of PTT markers PD-1, TIGIT and TIM3 in effector containing subsets γδ T cells, αβ T cells and NK cells from normal and tumor samples. **f**. Dot plot showing frequency of PTT triple positive Vδ2- T cells in normal and tumor samples. Significance assessed by one-sided Mann-Whitney test. Created with BioRender.com.

To define a comprehensive immunophenotyping of γδ T cells in cancer, we performed high-parameter flow cytometry (Fig. 1a) on cells from six resected renal cell carcinomas (RCCs) and compared them with data from tissues derived from five healthy donors (eight lymph nodes (LN), five spleens, five bone marrow samples, four peripheral blood samples and four lungs). Using force-directed plots where Vδ2 TCR expression was the main clustering driver (Fig. 1b), we identified populations particularly enriched in the tumors (Fig. 1c). After downsampling according to phenotypic similarity of the cells within individual samples, we identified 17 phenotypically distinct populations (Fig. 1d). Vδ2 TCR expression resolved 3 Vδ2^+^ clusters and 14 Vδ2^−^ clusters, suggesting lower phenotypic diversity in the Vδ2^+^ population compared with Vδ2^−^ cells. We also found that, while several clusters could be found in multiple compartments, cluster 15 was specifically enriched within the tumor, constituting almost half of the total tumor-infiltrated Vδ2^−^ cells (Fig. 1e). Interestingly, our flow data indicated a significant enrichment in Vδ2^−^ T cells in tumor versus normal kidney tissue (Extended Data Fig. 1d). Cluster 15 presented a relatively high expression of antitumor reactivity markers such as PD-1 or CD27 (Fig. 1f).

**Fig. 2:**
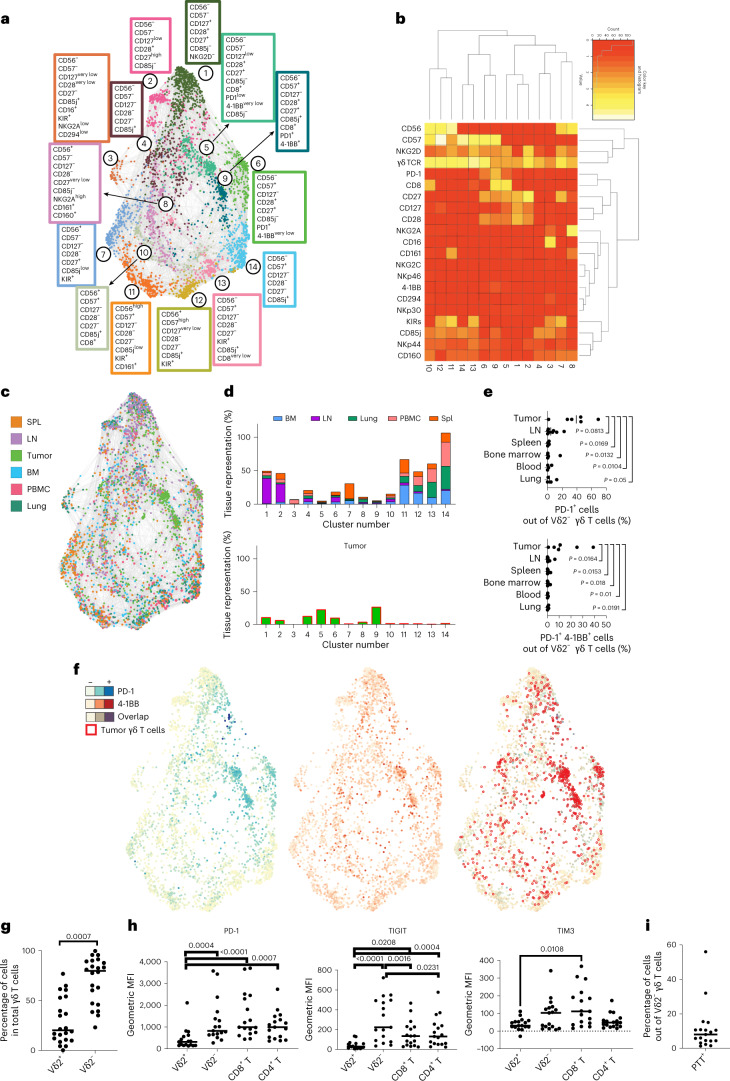
Tumors are enriched with Vδ2^−^ cells expressing PD-1 and 4-1BB. **a**–**i**, Vδ2^−^ cells from five healthy donor-derived LN, spleens (SPL), bone marrows (BM), lungs and PBMC samples and from patient-derived RCC tumors were analyzed by high-parameter flow cytometry. **a**, Force-directed graphs of Vδ2^−^ cells are shown. Colors correspond to 14 phenotypically distinct clusters. Enriched markers for each cluster are highlighted in color-matched text boxes. **b**, Heat map showing the differential expression of surface markers used for clustering. Dendrograms show the results of hierarchical clustering, used to group similar clusters (columns) and markers with similar cluster distributions (rows). **c**, Force-directed graph colored by source tissue type. **d**, Bar plots showing per tissue total cell frequencies of each phenotypic cluster in nontumor tissues (top) and tumor (bottom). **e**, Frequency quantification by flow cytometry of PD-1^+^ and PD-1^+^4-1BB^+^Vδ2^−^ T cells in five tumor samples and healthy tissues. Statistics were calculated by one-way ANOVA on log_10_-transformed data to account for differential distribution. **f**, Expression of PD-1 (blue, left) and 4-1BB (red, middle) across the different nodes and clusters is shown. The right panel displays the overlap between nodes positive for 4-1BB and PD-1 (maroon) superimposed with the distribution of the nodes from tumor samples (empty red circles). **g**, Frequency of tumor-infiltrating Vδ2^+^ and Vδ2^−^ T cells within the γδ^+^ T-cell compartment in 22 independent RCC tumor samples. *P* values obtained from Student’s *t*-test. **h**, Surface expression of PD-1, TIGIT and TIM3 measured by flow cytometry and depicted as geometric MFI on tumor-infiltrating Vδ2^+^, Vδ2^−^, CD8^+^ or CD4^+^ T cells. *P* values obtained by one-way ANOVA adjusted for multiple comparisons. **i**, Frequency of tumor-infiltrating Vδ2^−^ γδ^+^ T cells co-expressing PD-1, TIGIT and TIM3 (PTT^+^).

**Extended Data Fig. 2:**
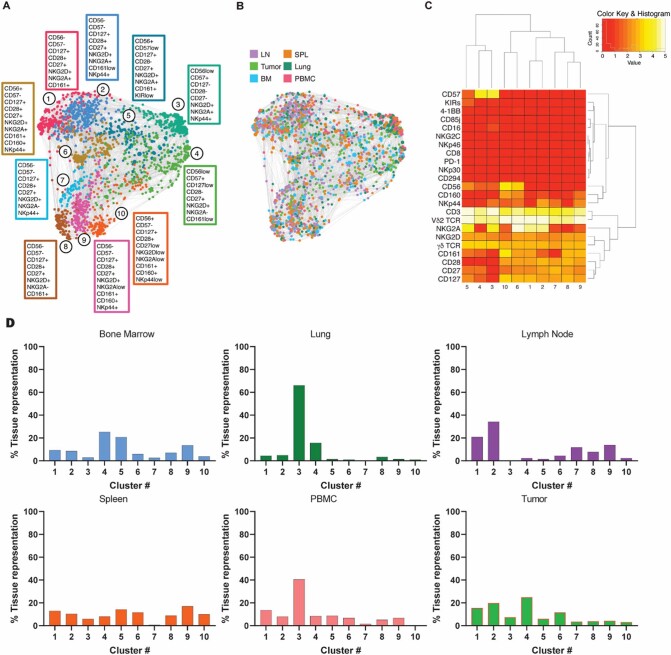
Vδ2+ T cell phenotypic clusters. **a**. Force-directed graphs of γδ Vδ2+ T cells are shown. Annotation of phenotypically similar nodes (clusters) are highlighted on force-directed graphs with the same color. Each color corresponds to a phenotypically distinct cluster (10 clusters total). Enriched markers for each cluster are highlighted in color-matched text boxes. **b**. Force-directed graph colored by tissue source is shown. **c**. Heatmap showing the differential expression of surface markers used for clustering. Dendrograms show the results of hierarchical clustering used to group similar clusters (columns) and markers with similar cluster distributions (rows). **d**. Bar plots showing per tissue total cell frequencies of each phenotypic cluster.

Given the marked differences between the Vδ2^+^ and Vδ2^−^ populations, we further analyzed them separately. In the subsequent clustering process, the cluster drivers for both Vδ2^+^ and Vδ2^−^ populations were markers linked to memory differentiation (CD28, CD27, CD56 and CD57) (refs. ^35,36^) (Fig. 2a). For Vδ2^+^ cells, CD57 was the main driver and showed a spatial enrichment of memory cells in the lung (dark green) from the less differentiated cells in the LN (violet). Despite the identification of ten phenotypically distinct populations, Vδ2^+^ cells showed a broad spatial distribution without being specifically enriched in the tumors (Extended Data Fig. 2).

In contrast, Vδ2^−^ cells at varying differentiation states were unevenly distributed between tissues (Fig. 2a). LN were enriched with ‘naïve/central memory’ cells (CD27^+^CD28^+^), spleens with effector cells (CD27^−/+^CD56^+^), PBMCs and lungs with terminally effector cells (CD56^+/−^CD57^+^) and bone marrow with effector and terminally differentiated effector clusters (Fig. 2b). Tumor tissues, however, were particularly enriched in clusters 5 and 9, accounting for almost half of all the tumor-infiltrating Vδ2^−^ T lymphocytes, with close-to-negligible representation in nontumor tissues (Fig. 2c,d).

**Extended Data Fig. 3:**
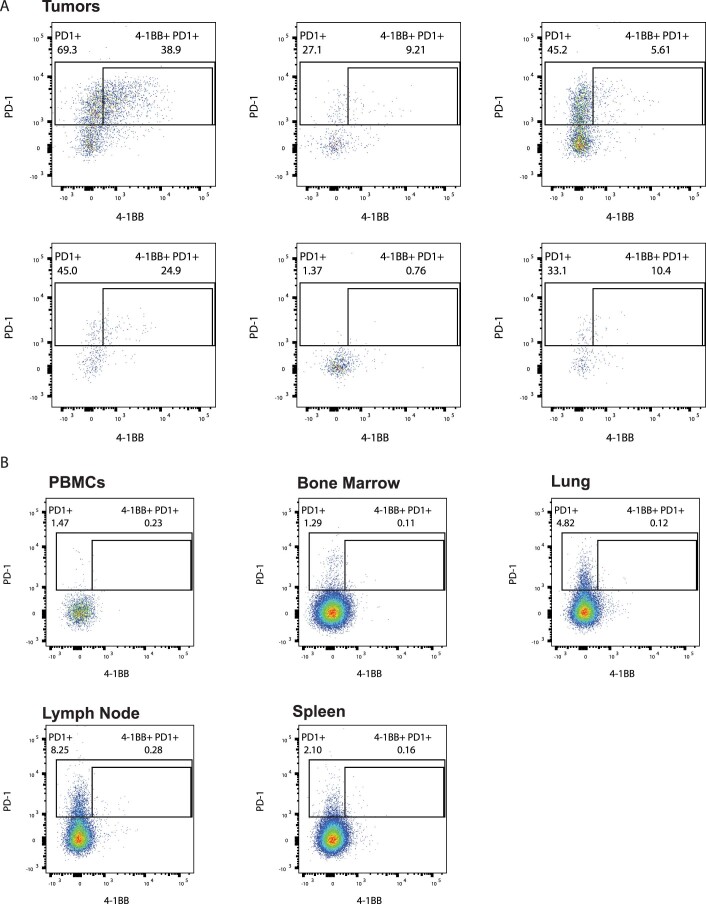
Tumor-infiltrating Vδ2- T cell subsets co-expressing 4-1BB and PD-1 are absent in healthy tissues. **a**. Representative flow cytometry dot plots from 6 different RCC patient tumor samples showing expression of PD-1 and 4-1BB in previously gated Vδ2- γδ T cells. Gates were used for data shown in Fig. 2e. **b**. Flow cytometry dot plots from healthy donor tissues representative sample showing expression of PD-1 and 4-1BB in previously gated Vδ2- γδ T cells. Gates were used for data shown in Fig. 2e.

These three populations were CD28^+^ and CD27^+^ with variable CD57 expression, but also expressed PD-1 and 4-1BB, two markers associated with T-cell activation and antigen experience in αβ T cells, respectively (Fig. 2a,f). Other Vδ2^−^ cells expressing PD-1 alone or with 4-1BB (Extended Data Fig. 3) were also significantly enriched in tumors compared with spleen, bone marrow and blood. Co-expression of PD-1 and 4-1BB was predominantly restricted to tumor-infiltrating cells compared with all analyzed tissues (Fig. 2e,f). Taken together, our investigation suggests that, whereas Vδ2^+^ cells are phenotypically homogeneous between healthy and tumor tissues, a portion of tumor-infiltrating Vδ2^−^ cells present a unique phenotype involving the expression of markers associated to both early activated (CD28 and CD27) and experienced effector cells (CD57, PD-1 and 4-1BB).

### Tumor Vδ2^−^ cells are functional while expressing inhibitory receptors

**Extended Data Fig. 4:**
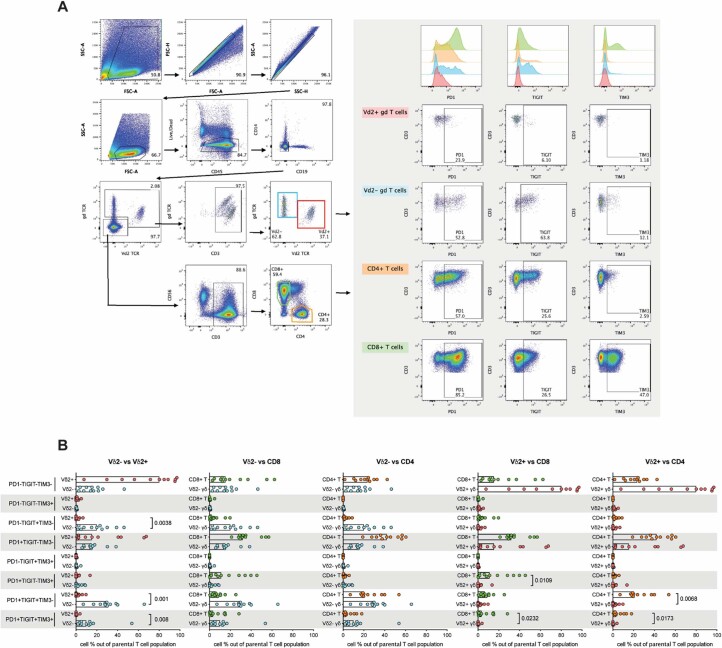
PD-1, TIGIT, and TIM-3 expression in lymphocyte populations from RCC patient samples by flow cytometry. **a**. Gating strategy in flow cytometry analysis of RCC samples (left). Mode-normalized histograms and dot plots showing expression of PD-1, TIGIT, and TIM-3 in αβ and γδ T cells (right). **b**. Boolean gating of PD-1, TIGIT, and TIM-3 expression in one-to-one comparisons between αβ and γδ T cell populations shown as percentage of total T cells from flow cytometry datasets. Each dot represents one patient sample. One-sided Student’s t-test statistical analyses for 2-column comparisons were applied. Bars represent the median.

**Extended Data Fig. 5:**
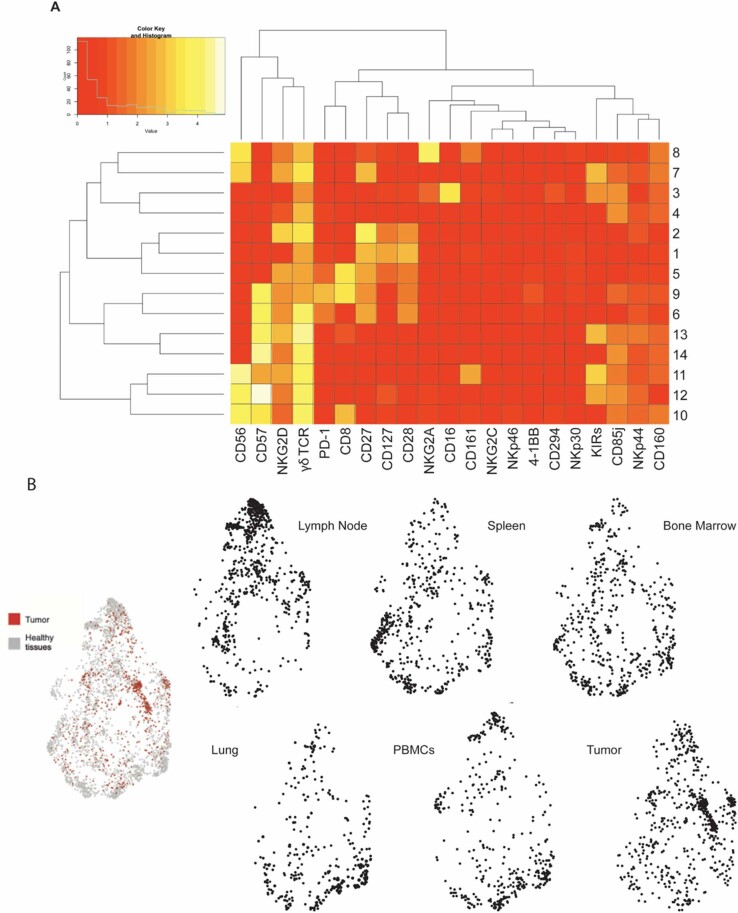
Vδ2- T cell tissue distribution. **a**. Heatmap showing the differential expression of surface markers used for clustering of Vδ2- T cells. Dendrograms show the results of hierarchical clustering, used to group similar clusters (rows) and markers with similar cluster distributions (columns). **b**. Node distribution of Vδ2- T cells from tumor (red) and healthy tissues (grey) in a force-directed graph. Force-directed graphs of Vδ2- T cells by source tissue type.

After observing the expression of PD-1 by a subset of tumor-infiltrating Vδ2^−^ cells, we focused on whether these cells possessed other markers of T-cell exhaustion. Because our overall analysis of 22 total donors revealed that Vδ2^−^ cells constituted the major γδ T-cell-infiltrating subset in RCC tumors, we separated γδ T cells into Vδ2^+^ and Vδ2^−^ subsets (Fig. 2g). We investigated expression of PD-1, TIGIT and TIM3 by flow cytometry, as these surface markers identify some of the most dysfunctional subsets in αβ T cells^37–39^. Intratumoral Vδ2^+^ cells expressed significantly lower levels of PD-1 and TIGIT compared with Vδ2^−^, αβ CD4^+^ and αβ CD8^+^ T cells. TIM3 levels were significantly lower in Vδ2^+^ cells only compared with αβ CD8^+^ T cells; nevertheless, Vδ2^+^ cells showed a clear trend of lower expression in comparison with Vδ2^−^ and αβ CD4^+^ T cells for this marker. On the other hand, expression of PD-1 and TIM3 on Vδ2^−^ cells was comparable to CD4^+^ and CD8^+^ αβ T cells, and surprisingly, TIGIT expression on Vδ2^−^ cells was higher than observed for both CD4^+^ and CD8^+^ αβ T cells (Fig. 2h and summarized data in Extended Data Figs. 4 and 5). We observed an apparent bimodal expression of TIGIT and TIM3 in Vδ2^−^ T cells. These data confirm that tumor-infiltrating Vδ2- cells express not only PD-1 but also other checkpoint receptors that are associated with T-cell dysfunction. Additionally, this analysis indicates that Vδ2^−^ cells are the main population of cells expressing PD-1, TIGIT and TIM3 within the γδ T-cell compartment.

**Fig. 3:**
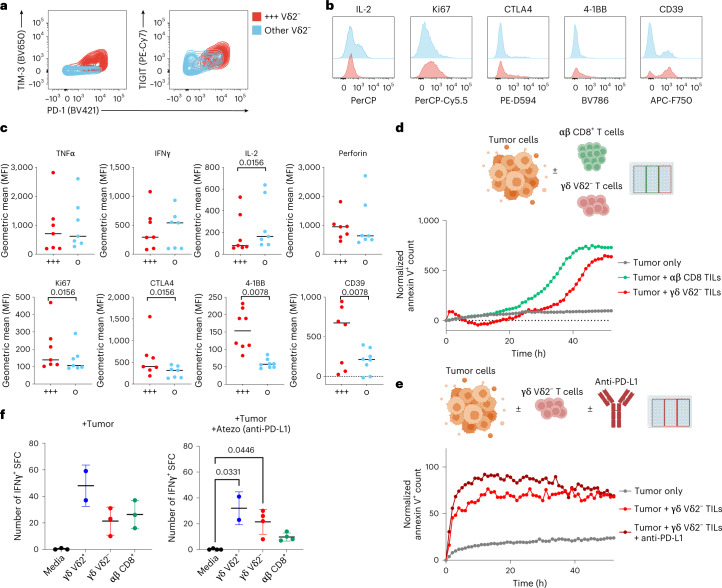
A mixed functional phenotype does not compromise cytotoxicity and cytokine release. **a**, Representative flow cytometry contour plots showing median values of surface expression of PD-1, TIGIT and TIM3 by tumor-infiltrating Vδ2^−^ γδ^+^ T cells in *n* = 8 tumor samples. Cells co-expressing PD-1, TIGIT and TIM3 (PTT^+^) are highlighted in red, while the remaining Vδ2^−^ cells are in blue. **b**, Flow cytometry histograms illustrating the differential expression of IL-2, Ki67, CTLA-4, 4-1BB, and CD39 on PTT^+^ Vδ2^−^ (in red) compared with non-PTT^+^ Vδ2^−^ cells (in blue). Cells were stimulated in vitro for 4 h with PMA+ ionomycin. **c**, Intracellular expression levels of TNF, IFNγ, IL-2, perforin and CTLA-4, and surface expression for Ki67, 4-1BB and CD39 were measured by flow cytometry and depicted by geometric MFI. *P* values by Student’s *t*-test are shown beneath each marker name. **d**, Representative time-course tumor cell killing (*n* = 2 independent experiments with different donors) in cultures of primary RCC tumor cells cultured alone (gray) or with autologous sorted αβ CD8^+^ (green) or γδ Vδ2^−^ TILs (red) (E:T 40:1). Quantification was performed by time-lapse microscopy. Target tumor cells were prestained with CytoLight Red, and tumor cell death was measured with annexin V^+^ staining of CytoLight Red^+^ cells. Values at time 0 from cultures with TILs alone were subtracted for normalization. **e**, Tumor cell killing in cultures with autologous Vδ2^−^ TILs with or without anti-PD-L1 (showing one of two independent experiments). **f**, IFNγ^+^ ELISpot counts from ex vivo cultures (*n* = 2 independent experiments with *n* = 2 different donors) with γδ or αβ sorted TILs stimulated with autologous kidney tumor cells (E:T 2:1) in the presence or absence of anti-PD-L1. Error bars represent standard deviation. *P* values obtained by nonparametric one-way ANOVA adjusted for multiple comparisons. Elements in **d** and **e** were created with http://BioRender.com.

**Extended Data Fig. 6:**
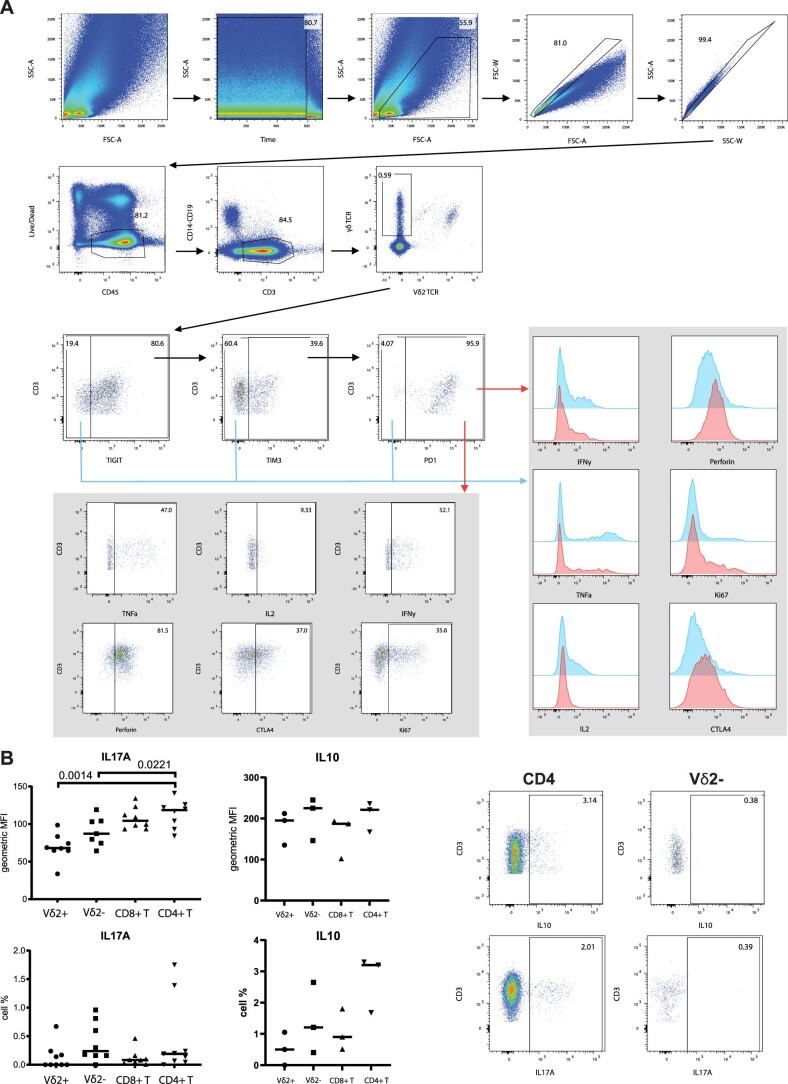
Vδ2- PTT+ T cells from RCC samples express effector molecules and low levels of IL-10 or IL-17A. **a**. Gating strategy in flow cytometry analysis of intracellular expression of effector molecules in Vδ2- PTT+ (red) and PTT- (blue) cells from n = 8 RCC samples. Histograms showing mean fluorescence intensity (MFI) were normalized to mode. **b**. Expression of IL-17A and IL-10 in αβ and γδ T cells from RCC samples after *ex vivo* stimulation (4 hrs). On the left panel, MFI (up) and percentage of total T cells (down) are shown. One-way ANOVA analysis was performed. On the right panel, representative dot plots showing expression of regulatory cytokines IL-10 and IL-17A in CD4 αβ T cells and Vδ2- T cells from RCC tumor patient samples.

**Extended Data Fig. 7:**
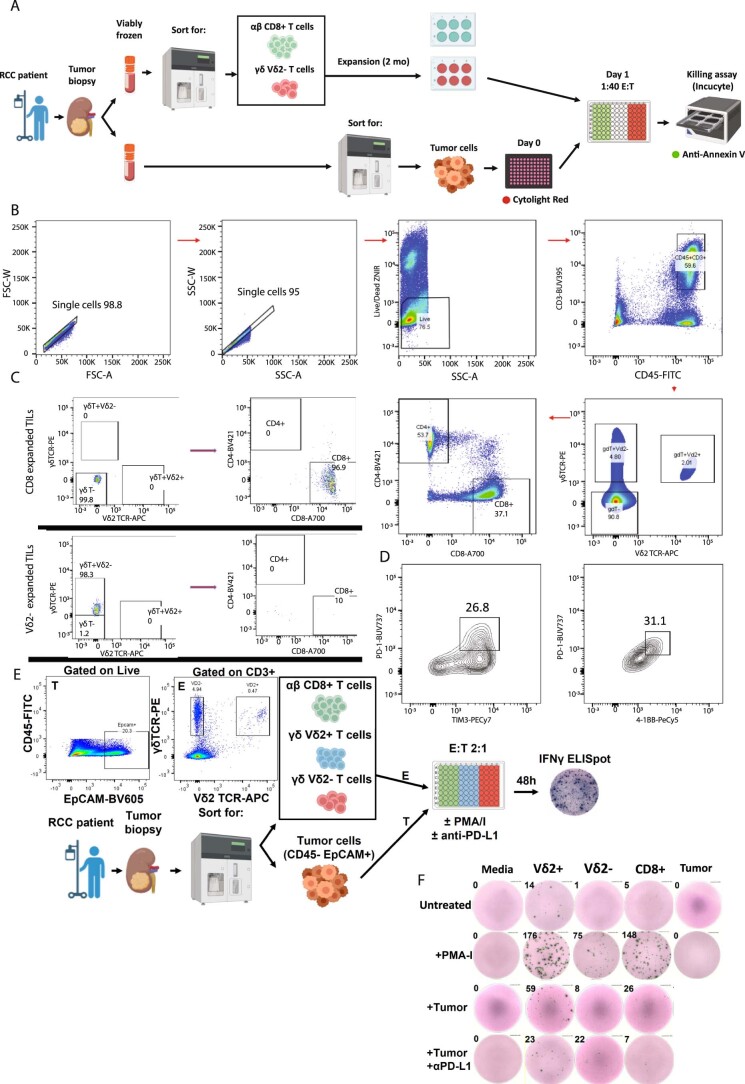
Autologous killing assay with sorted lymphocytes. **a**. Diagram showing the workflow to obtain pure tumor cell, αβ or γδ T cell populations from RCC biopsies and conditions for the autologous killing assay co-cultures. **b**. Flow cytometry gating strategy used to sort TIL populations from digested RCC tumor biopsies. **c**. Flow cytometry dot plots showing the sorted αβ CD8+ or γδ TIL populations after downstream expansion before adding them to cytotoxicity assays in co-culture with target autologous tumor cells. **d**. Flow cytometry contour plots showing expression of PD-1, TIM3 and 4-1BB in expanded Vδ2- T cells used in autologous killing assays. **e**. Diagram showing the workflow for generating fresh autologous TIL:tumor cocultures for immune stimulation and readout by ELISpot. Pseudocolor flow cytometry plots show gating strategy for cell sorting of target populations. **f**. Human IFNγ ELISpot representative individual well images from TIL:tumor cocultures with QC-passed counts shown on the upper left edge of each well. Row titles stand for stimulation conditions. Column titles show sorted cell populations present in the wells. Created with BioRender.com.

Co-expression of multiple checkpoint receptors is usually linked to reduced secretion of effector molecules and is considered a clear sign of functional exhaustion on classical αβ T cells^38^. Drawing from such knowledge, we examined whether Vδ2^−^ cells co-expressed multiple checkpoints and whether this was linked to reduced secretion of effector molecules. Hence, we confirmed the presence of Vδ2^−^ cells co-expressing PD-1, TIGIT and TIM3, which overall represented about 8% of tumor-infiltrating Vδ2^−^ cells (Fig. 2i) and which were enriched in tumor versus healthy kidney samples (Extended Data Fig. 1e). We designated this population PTT^+^, for PD-1^+^TIGIT^+^TIM3^+^ (Fig. 3a). Interestingly, PTT^+^ cells expressed lower amounts of IL-2 than nonexhausted cells upon ex vivo restimulation (Fig. 3b,c), while no significant changes were observed for the release of IFNγ, TNF and perforin. Additionally, PTT^+^ cells showed significantly higher levels of CTLA-4, Ki67, 4-1BB and CD39, all of which are expected to be expressed in antigen-experienced cells^40^. Vδ2^−^ cells showed equal or lower levels of previously reported immunosuppressive IL-10 and IL17A (Extended Data Fig. 6). To confirm their cytotoxic potential, we co-cultured isolated tumor cells from resected RCC tumors with expanded autologous Vδ2^−^ T cells, containing the PTT^+^ population, or αβ CD8^+^ tumor-infiltrating lymphoyctes (TILs), and quantitated killing through time-lapse microscopy (summarized in Extended Data Fig. 7). Expanded Vδ2^−^ cells retaining expression of PD-1, 4-1BB and TIM3 (Extended Data Fig. 7) were able to kill autologous tumor cells at comparable levels and kinetics to αβ CD8^+^ T cells (Fig. 3d). This cytotoxicity was nonsignificantly enhanced in vitro in the presence of anti-PD-L1 (Fig. 3e). To confirm tumor reactivity without ex vivo expansion, we performed co-culture experiments with freshly sorted TILs with autologous malignant cells. Vδ2^+^ cells showed a nonsignificantly increased ability to secrete IFNγ compared with Vδ2^−^ or CD8. Notably, in the presence of anti-PD-L1, both Vδ2^−^ and Vδ2^+^ showed comparable spot-forming counts, superior to wells containing CD8:tumor co-cultures (Fig. 3f and further detailed in Extended Data Fig. 7). Vδ2^−^ production of IFNγ was not modulated by anti-PD-L1. All together, these data show that PTT^+^ Vδ2^−^ cells possess an ‘exhausted’ phenotype in which they maintain minimally blunted effector function when infiltrating the tumor microenvironment.

#### Exhausted Vδ2^−^ cells maintain expression of effector molecules

**Fig. 4:**
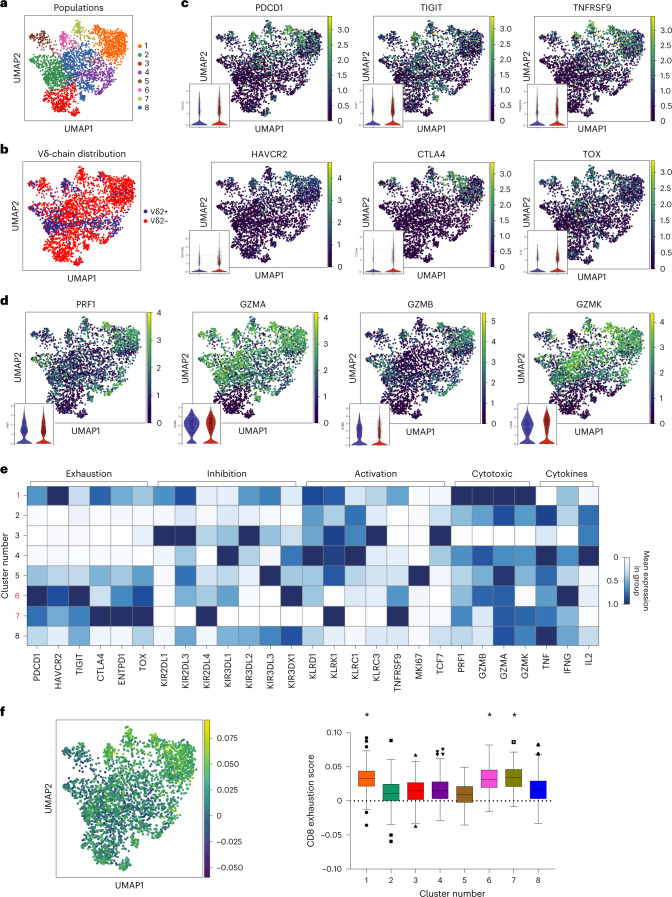
Coexisting exhaustion and effector phenotypes and broad γδ TCR usage. **a**, UMAP plot of 3,024 sorted γδ T cells obtained from RCC tumors (*n* = 6 patients). Phenotypically distinct clusters are represented with different colors. **b**, UMAP plot colored by TCR Vδ2 usage, based on Vδ gene expression. Vδ2^+^ cells are marked in blue, while Vδ2^−^ cells are marked in red. **c**, Relative expression of select T-cell exhaustion markers, with violin plots split by Vδ2 usage in bottom left corner of each UMAP. **d**, Relative expression of select effector function markers, with violin plots split by Vδ2 usage in bottom left corner of each UMAP. **e**, Heat map showing the relative expression of select markers of exhaustion, inhibition, activation, cytotoxic potential and cytokine secretion for each cluster shown in **a**. **f**, Left: UMAP plot colored by gene set score of the exhausted CD8 αβ T-cell gene signature described by Scott et al.^41^. Of the 1,661 identified genes, 1,327 genes overlapping with our dataset were used to calculate the gene score on the 3,024 sorted γδ T cells. Right: box plot shows CD8 exhaustion score split by phenotypic cluster shown in **a**. Each box represents 0.25–0.75 percentile of the exhaustion signature score. Horizontal line represents the median. Error bars and outliers determined by one-way Tukey’s statistical test. *P < 0.05 versus clusters 2, 3, 4, 5 and 8.

To further dissect the functional states of the γδ T cells, we performed single-cell RNA sequencing (scRNA-seq) on flow-cytometry-sorted γδ T cells from six human RCC tumors and analyzed the resulting data using Scanpy (Single-Cell Analysis in Python package). After standard quality control and preprocessing, cells were clustered with the Leiden algorithm, resulting in eight independent γδ subsets (Fig. 4a). Vδ2^+^ and Vδ2^−^ cells were identified on the basis of their Vδ gene expression (Fig. 4b). Interestingly, scRNA-seq analysis revealed clusters enriched predominantly with Vδ2^+^ (2 and 4) or Vδ2^−^ cells (1, 3, 7 and 8) as well as hybrid clusters (5 and 6), further supporting the observation that these two subpopulations of γδ T cells can be found in distinct states and show substantial differences in function (Fig. 4c). We confirmed the expression of several immune checkpoints PD-1 (*PDCD1*), *TIGIT*, TIM-3 (*HAVCR2*), *CTLA4* and 4-1BB (*TNFSRF9*), but also effector molecules (granzymes and perforin) in γδ T-cell subsets (Fig. 4c,d). Both Vδ2^+^ and Vδ2^−^ T cells expressed messenger RNA for checkpoint receptors, although subsets enriched in Vδ2^−^ were more prevalently positive for checkpoints. Of note, in our dataset, Vδ2^+^ cells were the main and almost exclusive contributors to perforin and granzyme B expression, whereas Vδ2^−^ cells showed higher granzyme A and granzyme K expression (Fig. 4d). We identified clusters 1, 6 and 7 (all containing Vδ2^−^ cells in varying abundance) as high expressors of *PDCD1*, *TIGIT* and *HAVCR2*, thereby resembling the PTT^+^ phenotype, accompanied by moderately high expression levels of cytotoxic enzymes including GZMB or PRF1, particularly in cluster 1, and low expression of the progenitor marker *TCF7* (Fig. 4e)*. TCF7* expression was mainly confined to the Vδ2^−^ cluster 3. Vδ2^+^-dominated clusters 2 and 4 did not show signs of exhaustion. Looking at expression of innate receptors, Vδ2^−^ clusters 1 and 7 showed high levels of NKG2D (*KLRK1*), while differing in the expression of others. Hybrid cluster 6 showed a radically different innate expression pattern, with highest levels for *KIR3DX1*. TCF1-expressing cluster 3 co-expressed several inhibitory innate receptors such as *KIR2DL1*, *KIR2DL3*, *KIR3DL2* or *KLRC3*. Overall, the innate receptor landscape appeared intriguingly diverse. These results support our observations obtained via flow cytometry showing that Vδ2^−^ cells have the potential to express immune checkpoints and at the same time retain their effector molecules and other inhibitory and activating receptors, probably indicating a diverse functional spectrum in the different γδ T-cell populations, irrespective of their exhaustion status.

Because various subsets of tumor-infiltrating Vδ2^−^ cells co-expressed several immune checkpoints and showed a reduced capacity to express IFNγ, we questioned how γδ T cells in our analysis resembled exhausted CD8^+^ αβ T cells as defined by a gene signature described by others^41^. When applying this signature to our γδ T-cell dataset, cells in clusters 1, 6 and 7 from Fig. 4a showed the highest ‘exhaustion score’ (Fig. 4f), validating our previous analysis and confirming that subsets of γδ T cells resembled canonically exhausted CD8^+^ αβ T cells despite retaining the expression of several effector molecules. Together, these observations suggest functional capacity for ‘exhausted’ diverse tumor-infiltrating γδ T-cell subsets.

#### Expansion of γδ cells is associated with exhaustion but not Vδ usage

**Fig. 5:**
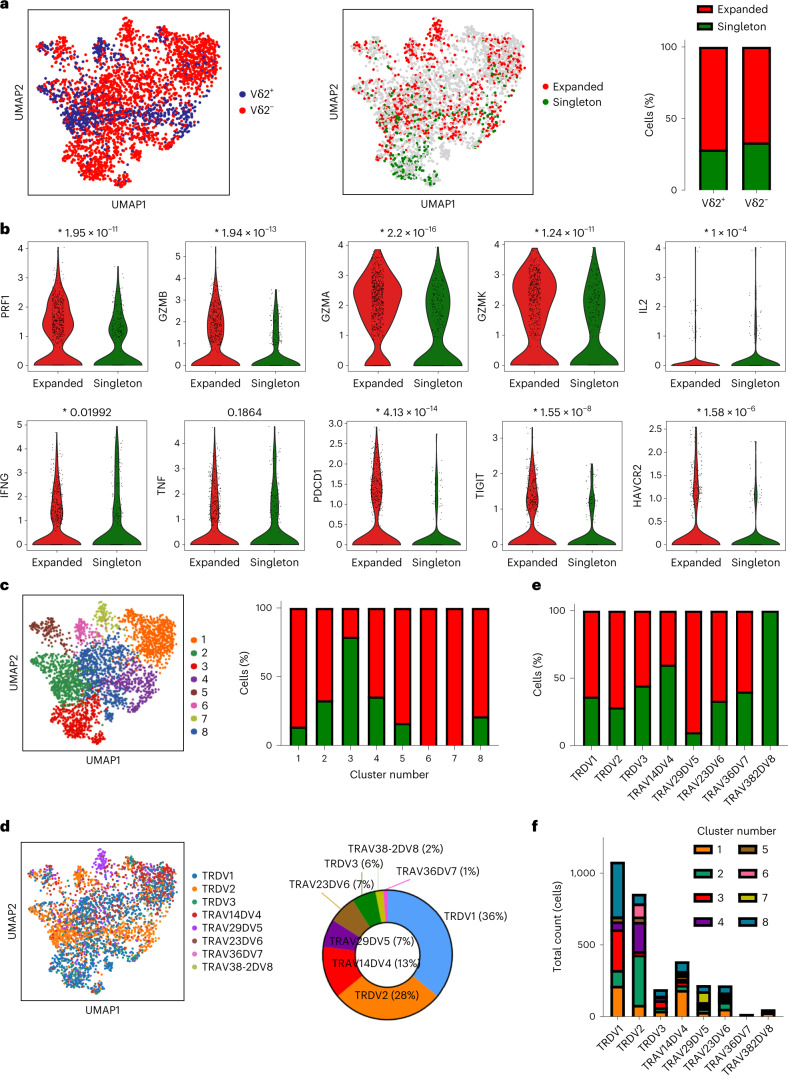
TCR expansion of exhausted γδ T-cell clusters with no Vδ chain bias. **a**, Left: UMAP plot of 3,024 sorted γδ T cells from six patients with RCC colored by TCR Vδ2 usage, based on Vδ gene expression. Vδ2^+^ cells are marked in blue, while Vδ2^−^ cells are marked in red. Middle: UMAP plot showing 1,033 annotated TCRs colored by degree of expansion. Expanded (more than one copy detected) cells are marked in red, while singletons or nonexpanded cells are marked in green. Right, stacked bar plot showing γδ TCR expansion based on Vδ2 chain usage. **b**, Violin plots showing expression of several phenotypic markers in expanded and nonexpanded γδ T cells. *P* values are displayed on top of each plot. **c**, Left: UMAP plot showing phenotypically distinct clusters represented with different colors. Right: stacked bar plot showing γδ TCR expansion for each phenotypic cluster. **d**, Left: UMAP plot colored by TRDV gene expression. Right: donut plot showing relative representation of each γδ TCR in the dataset. **e**, Stacked bar plot showing γδ TCR expansion based on γδ TCR usage. **f**, Stacked bar plot showing total cell counts by γδ TCR usage, colored by phenotypic clusters.

**Extended Data Fig. 8:**
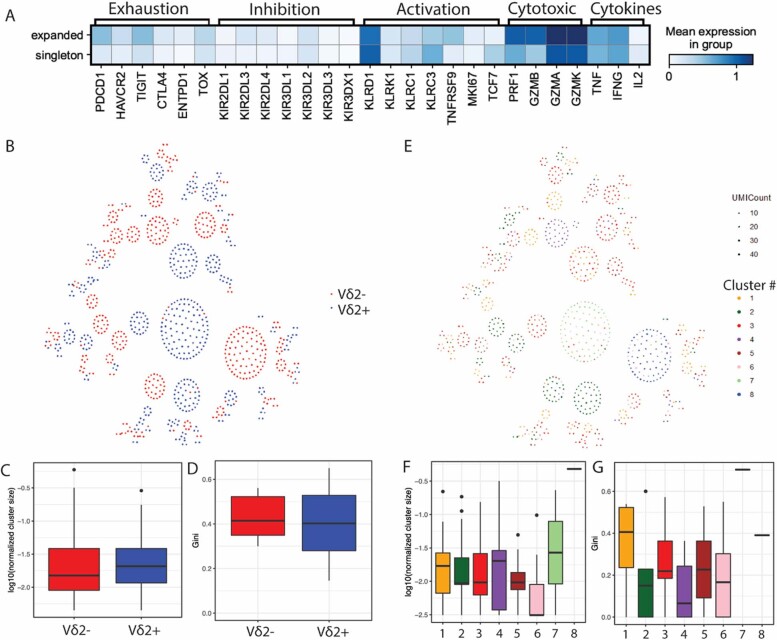
γδ TCR analyses of clonal expansion and diversity. **a**. Heatmap showing the relative expression of select markers from T cell clonotypes that were either expanded (>1) or singleton cells shown in Fig. 5a (middle). **b**. Social network diagram showing all expanded γδ TCR clonotypes, colored by Vδ chain usage. Each dot represents an individual cell. Each node of dots clustered together represents cells with an identical TCR. **c**. Network analysis was done for each patient (n = 6) and cluster size represents total number of cells sharing a given clone by Vδ2- (red) or Vδ2+ (blue). The log-normalized cluster sizes are shown. **d**. The clonal diversity (gini index) was calculated based on the number of cells belong to a single TCR clone within for each patient by Vδ2- (red) or Vδ2+ (blue). **e**. Social network diagram showing all expanded γδ TCR clonotypes, colored by phenotypic cluster. Each dot represents an individual cell. Each node of dots clustered together represents cells with an identical TCR. Network analysis was done for each patient and cluster size represents total number of cells sharing a given clone by phenotypic clusters. **g**. The clonal diversity (Gini index) which was calculated based on the number of cells belong to a single TCR clone within for each patient by phenotypic clusters. In all boxplots, each box represents 0.25–0.75 percentile of the transformed cluster size or Gini score, with line extensions up to 1.5 times the interquartile range; horizontal line represents the median.

We analyzed TCR expansion in our dataset; we found more expanded than nonexpanded γδ T cells, the latter referred to as ‘singletons’. Vδ2^−^ cells showed comparable degree of TCR expansion to Vδ2^+^ cells, with 59% and 65% of expanded cells, respectively (Fig. 5a). Expanded cells possessed significantly higher expression of exhaustion marker genes including *PDCD1*, *TIGIT* and *TNFRSF9*, but also expressed effector genes including *IFNG*, *PRF1* and several granzymes, and a trend for increased *TNFA* (Fig. 5b). There was no significant difference in innate receptor expression in expanded versus singleton cells (Extended Data Fig. 8a), probably due to the Vδ-chain heterogeneity within expanded cells. Vδ2^+^ and Vδ2^−^ cells had equivalent levels of clonal expansion (Extended Data Fig. 8b,c) and TCR diversity (Extended Data Fig. 8d). Exhausted Vδ2^−^ dominated clusters 1 and 7, together with hybrid cluster 6, were the most expanded (Fig. 5c). Cluster 3, the Vδ2^−^ dominated population expressing high levels of the progenitor gene *TCF7* (Fig. 4e), showed very low expansion. When analyzing the data by TCR chain usage, Vδ1 (*TRDV1*) were the most prevalent among Vδ2^−^ T cells with a 36% overall representation (50% of Vδ2^−^ T cells), followed by Vδ4 (*TRAV14DV4*, 13% overall, 18% within Vδ2^−^), Vδ5 (*TRAV29DV5*, 7% overall, 9.7% within Vδ2^−^), Vδ6 (*TRAV23DV6*, 7% overall, 9.7% within Vδ2^−^) and Vδ3 (*TRDV3*, 6% overall, 8.3% within Vδ2^−^) (Fig. 5d). When looking at Vδ-chain specific TCR expansion, Vδ5 showed the highest expansion (89%), probably owing to its predominance in cluster 7. Most Vδ2^−^ subsets showed comparable degrees of expansion ranging from 40% to 60%, with Vδ1 at 55.6% (Fig. 5e).

Next, we examined associations between the phenotypic populations and Vδ-chain usage in our dataset. We confirmed that Vδ2^+^ cells were primarily represented in clusters 2 and 4. Exhausted and expanded cluster 1 showed a broad Vδ2^−^ distribution. Cluster 6 was predominantly Vδ2, but also included some Vδ4 and Vδ6 clonotypes. The final ‘PTT^+^’ population, cluster 7, was enriched for Vδ5 (Fig. 5f). Clonal expansion and TCR diversity were similar across the different populations, although there was a trend for increased expansion cluster size for clusters 1 and 7 (Extended Data Fig. 8e–g). These data highlight the relevance of unbiased Vδ2^−^ TCR expansion in RCC, pointing to the presence of potentially antitumoral phenotypes beyond previously characterized Vδ1^−^ tumor infiltrating T cells.

#### A Vδ2^−^ signature correlates with clinical benefit in PD-L1 blockade

**Fig. 6:**
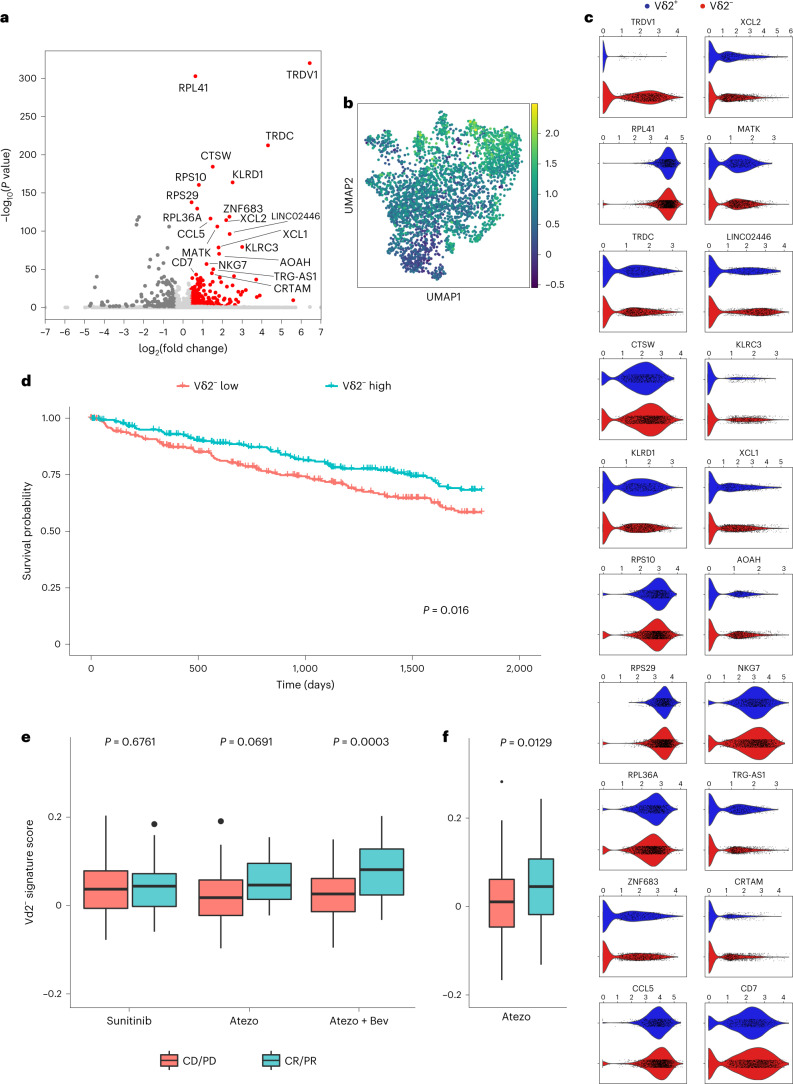
Transcriptional signature of Vδ2^−^ γδ T cells associates with favorable clinical outcomes. **a**, Volcano plot showing differential gene expression of 2,117 Vδ2^−^ γδ T cells versus 901 Vδ2^+^ γδ T cells and 14,519 αβ CD4 and CD8 T cells. The 20 most upregulated genes by two-sided Wilcoxon rank-sum *P* value (labeled) make up the Vδ2^−^ gene signature. Genes with adjusted *P* value <0.05 and absolute log fold change >0.4 are colored in red. Nonsignificant outliers (adjusted *P* value >0.05) have been clipped to LFC (−7, 7). **b**, UMAP plot colored by the gene set score of the Vδ2^−^ gene signature, corresponding to their average raw expression subtracted by the average raw expression of a randomly sampled reference set of genes. **c**, Violin plots comparing the expression of the 20 genes in the Vδ2^−^ gene signature in Vδ2^−^ versus Vδ2^+^ γδ T cells. Significance was assessed using two-sided Wilcoxon rank-sum tests. **d**, Kaplan–Meier survival curves (5 years) of 534 patients with RCC from TCGA, split by above versus below median enrichment of the Vδ2^−^ gene signature within their bulk RNA data. **e**, Box plots showing enrichment scores for the Vδ2^−^ gene signature in bulk RNA data from pretreatment tumors of 263 patients (86 atezolizumab, 88 atezolizumab plus bevacizumab, and 89 sunitinib) with advanced RCC on the IMmotion150 clinical trial. Plots are split by clinical response (CR/PR) versus nonresponse (SD/PD) and grouped by treatment—sunitinib, atezolizumab, or atezolizumab in combination with bevacizumab. **f**, Box plots showing enrichment scores for the Vδ2^−^ gene signature in bulk RNA data from pretreatment tumors of 168 patients (84 each arm) with advanced urothelial bladder cancer on the IMvigor210 clinical trial. Plots are split by clinical response (CR/PR) versus nonresponse (SD/PD). All patients were treated with atezolizumab. In all box plots, each box represents 0.25–0.75 percentile of the gene signature score, with line extensions up to 1.5 times the interquartile range; horizontal line represents the median. CR, complete responder; PR, partial responder; SD, stable disease; PD, progressive disease; Vδ2^−^, negative for the TCR Vδ2 gene.

We derived a transcriptional signature from the tumor-infiltrating Vδ2^−^ cells by running a differential expression analysis comparing the Vδ2^−^ γδ cells with the remaining γδ T cells, and to CD4^+^ and CD8^+^ αβ T cells sorted from the same tumors (Fig. 6a; see gene expression list on Supplementary Table 1). We used the resulting Vδ2^−^ signature to calculate a ‘Vδ2^−^ score’ in our dataset (Fig. 6b). In this analysis, the highest Vδ2^−^ score located to clusters 1, 6 and 7 from Fig. 4a, all of them co-expressing *PDCD1*, *TIGIT* and *HAVCR2*. The Vδ2^−^ signature included, in addition to ubiquitous ribosomal proteins, differentially elevated co-expression of both inhibitory (*KLRD1*, *MATK* and *AOAH*) and effector genes (*NKG7*, *CRTAM* and *CCR5* ligand *CCL5*), as well as T-cell-attracting chemokines (*XCL1* and *XCL2*) or genes involved in the development of long-term tissue-resident innate effector cells such as *CD7* or *ZNF683* (ref. ^42^) (Fig. 6c).

To examine whether tumor-infiltrating Vδ2^−^ γδ T cells are clinically relevant, we first applied this transcriptional signature to the available tumor expression dataset in The Cancer Genome Atlas (TCGA) from patients with RCC. Remarkably, 5 year survival was significantly higher for patients with a higher Vδ2^−^ transcriptional score (Fig. 6d). Next, we examined whether tumor-infiltrating Vδ2^−^ γδ T cells could be associated with clinical response to PD-1 blockade by interrogating the tumor expression data in 263 patients from the IMmotion150 kidney cancer study NCT01984242 (ref. ^43^). In this trial, patients with metastatic RCC were treated with sunitinib, atezolizumab (anti-PD-L1) or atezolizumab + bevacizumab (anti-VEGF). Our Vδ2^−^ molecular signature significantly correlated with clinical responses in patients treated with the combination of atezolizumab + bevacizumab (Fig. 6e). We also observed a trend for improved responses in patients treated with atezolizumab alone compared with sunitinib. These findings were consistent with the described associations to a T-effector gene signature^5^. To examine whether tumor-infiltrating Vδ2^−^ γδ T cells might be relevant in PD-1 blockade in another disease, we interrogated the tumor expression data in 168 samples from the IMvigor210 bladder cancer trial NCT02108652 (ref. ^44^) with our Vδ2^−^ gene signature. In this trial, patients with locally advanced and metastatic urothelial carcinoma were treated with atezolizumab. Our Vδ2^−^ signature correlated with improved clinical responses in this trial (Fig. 6f). These data suggest a role of γδ T cells and specifically of Vδ2^−^ cells as being relevant agents in successful responses to ICI.

## Discussion

Development of γδ-based immunotherapies has relied preferably on Vδ2^+^ due to their convenient reactivity to phosphoantigens and better-described antibody-dependent Vδ2^+^ tumor cell killing^45^. They have been traditionally used in allogeneic adoptive cell immunotherapy clinical trials after ex vivo expansion or by inducing their proliferation in vivo^15,46^. In such trials, even though showing acceptable toxicities, partial or complete responses were relatively infrequent^12,47,48^, suggesting that either Vδ2^+^ were not involved in antitumor responses in these patients or that inhibitory mechanisms might be at play. The first option seems very unlikely, as Vδ2^+^ cells mediate strong cytotoxic activity against tumor cells in vitro and a few patients did obtain clinical benefit. This highlights the importance of tissue-specific analyses when dissecting γδ T-cell biology in the search for therapeutic strategies.

Non-Vδ2, and specifically Vδ1 cells, have previously been associated to tumor reactivity and clinical benefit in breast cancer^49,50^ and, more recently, lung cancer^51^. In this latter study, overall Vδ1 T-cell frequencies were not significantly different between tumor and healthy tissues. This later observation differs from our study in kidney cancer, where we observe a polarization towards enriched Vδ2^−^ in tumors versus healthy kidney tissue, in line with other studies showing Vδ2^−^ tumor reactivity in patients with colon cancer treated with checkpoint inhibition^52^. On the other hand, Wistuba and colleagues, among others, have shown the clinical relevance of bloodborne Vδ2 and not Vδ1 in response to anti-CTLA4 in patients with melanoma^53^, while in ovarian cancer, Foord and colleagues reported no apparent role in antitumor activity for Vδ1 T cells, which showed an exhausted-like phenotype and did not correlate with clinical benefit^54^. In the case of renal cancer, Inman and colleagues questioned the overall role of γδ in antitumor activity by showing no correlation between their frequency and any clinicopathological feature in stained archival samples^55^. In the case of liver cancer, both Vδ2^+^ and Vδ2^−^ cells showing a tissue-resident memory phenotype were associated to survival in a recent study led by Zakeri and colleagues. Importantly, αβ T cells did not correlate with clinical benefit in this study^56^. Features from γδ T cells, such as the high variability of the TCR repertoire in still not fully studied subsets like Vδ1, when compared with Vδ2 (ref. ^57^), might be crucial to determine the function of these tumor-infiltrating lymphocytes. Hence, on the basis of the recent literature, contribution of different γδ populations in antitumor immunity is controversial.

To our knowledge, this represents the first analysis to comprehensively immunophenotype γδ T cells residing in normal tissues and in renal cancer. Our analysis shows reduced tumor infiltration of non-activated Vδ2^+^ cells. In RCC, modest but promising results have encouraged new strategies aiming at effective infiltration of cytotoxic Vδ2^+^ γδ T cells^8–13^. Another strategy could be to leverage the cytotoxic potential of other γδ T-cell subsets. Our data suggest that specific populations of Vδ2^−^ cells may localize in certain tissues and exert specialized functions, a hypothesis that others have also proposed^58^. This proved to be particularly true for a subset of tumor-infiltrating Vδ2^−^ cells identified in our analysis, which we designated PTT^+^ owing to their co-expression of established markers of αβ T-cell exhaustion, PD-1, TIGIT and TIM-3. These cells comprised multiple Vδ2^−^ TCR gene and not just Vδ1 cells, indicating contributions by different γδ TCRs to tumor recognition. These PTT^+^ cells possessed an exhaustion gene signature described in the literature^41^ and lacked expression of TCF7, resembling terminally exhausted CD8^+^ αβ T cells. However, these Vδ2^−^ T cells also retained a significant level of effector function overall and were clonally expanded.

Other groups have reported PD-1-mediated regulation of γδ T cells^24,59^. Moreover, PD-1-expressing tumor-infiltrating γδ T cells have also been identified in neuroblastoma^29^, pancreatic ductal adenocarcinoma^30^, colorectal cancer^31^ and multiple myeloma-infiltrated bone marrow^32^. Reduced PD-1 levels have been observed in γδ T cells from peripheral blood from patients with remitting acute myeloid leukemia^60^. Although there is not extensive literature regarding the consequences in pathological conditions of γδ T-cell exhaustion, PD-1 upregulation in activated γδ T cells has been associated with reduced IFNγ secretion in leukemia patients^59^, reduced cytotoxicity against several PD-L1-expressing tumor cell lines^61^, and impaired αβ T-cell function^30^. Vδ2^−^ T cells are thought to be the predominant PD-1-expressing γδ T-cell population, but more extensive research is needed to fully elucidate their role in tumors. The PTT^+^ Vδ2^−^ expressed 4-1BB, CD39 and CTLA-4, consistent with previous encounters with cognate antigen and subsequent TCR activation^40,62^. Cells aligning to this phenotypic population are heterogeneously distributed in at least three distinct populations and are not restricted to the Vδ1 clonotype. In contrast to previous reports, PTT^+^ cells also maintained significant effector function^63,64^, such as higher Ki67, TNF, IFNγ, perforin, GZMA or GZMK, when compared with the non-PTT^+^ population. This alternative form of exhaustion, in combination with the cytotoxic potential of autologous Vδ2^−^ cells to kill tumor cells shown in this study, emphasizes the potential of Vδ2^−^ subsets as important mediators of antitumor responses, as others have also proposed^65–67^, and could lead to significant advances in adoptive cell therapies that have been shown sensitive to canonical αβ T-cell checkpoints^68^. Further research will be crucial to elucidate the contribution to tumor rejection from the PTT^+^ subsets, as well as to update controversial findings in which immunosuppressive or pro-tumorigenic γδ T-cell subsets have been described^21,28,30,69,70^. In this sense, the phenotypic diversity in innate receptor expression in γδ T cells described in this study (Fig. 4e) could point towards the unambiguous characterization of relevant ligand–receptor interactions modulating the tumor immune microenvironment.

**Extended Data Fig. 9:**
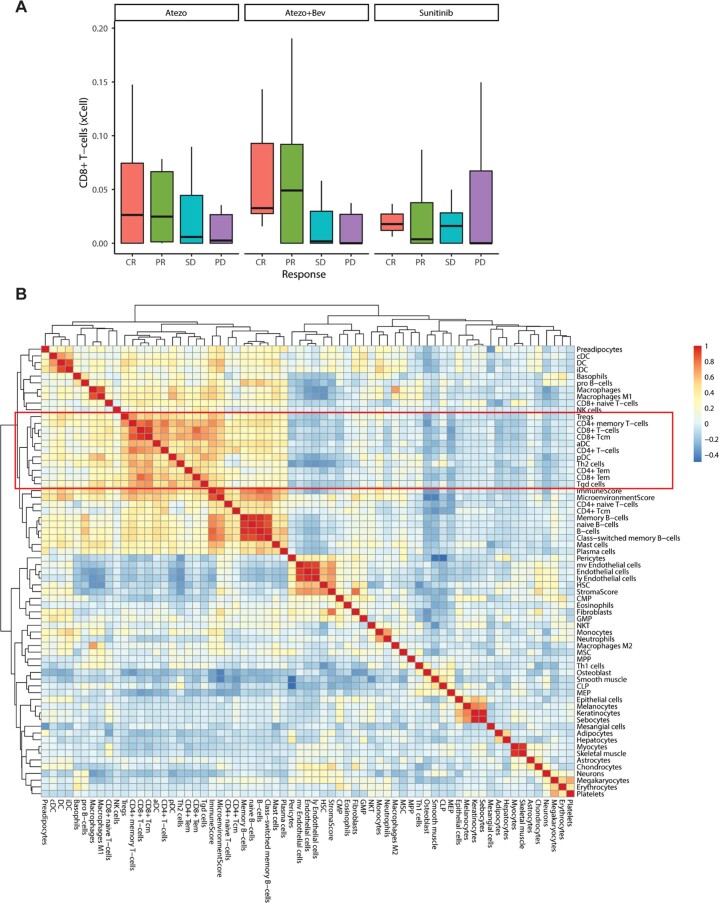
T cell presence in the tumor correlates with clinical response. **a**. Predicted frequency of CD8+ αβ T cells derived from bulk RNAseq data using xCell cell type enrichment analysis^64^, split by disease outcome and the trial arms of the IMmotion150 mRCC clinical trial involving n = 305 randomized patients. Each box represents 0.25–0.75 percentile of the CD8 frequency, with line extensions up to 1.5 times the interquartile range; horizontal line represents the median. **b**. Heatmap showing pairwise correlations of cell populations clustered by bulk RNAseq data from the Immotion150 clinical trial. All αβ and γδ T cell clusters are highlighted inside the red rectangle. Highest correlation (expressed as co-variation score) for γδ T cells is with CD8+ effector and central memory cells.

Remarkably, the gene signature derived from the Vδ2^−^ subset correlated with improved survival in kidney cancer patient data from the TCGA database, as well as with improved clinical response in patients with RCC and urothelial cancer treated with PD-1 blockade. Even though researchers found αβ CD8 T-cell effector and myeloid gene signatures correlated with progression-free survival in the IMmotion150 kidney trial^5^ (Extended Data Fig. 9), our results highlight the contribution of the γδ T-cell compartment to clinical benefit. The present study contributes to recent studies where γδ T cells have been identified as potential prognostic biomarkers associated with clinical benefit in a wide variety of malignancies^27,71^. Such studies have highlighted the importance of dissecting γδ-specific gene signatures associated with favorable clinical outcomes from broader ‘effector’ gene expression patterns^72^ in a tumor-agnostic fashion^27^. In that sense, our study reinforces the notion that exploring mixed gene expression programs in more detail, that is, simultaneous expression of exhaustion and cytotoxicity-associated genes like in the Vδ2^−^ signature or the PTT^+^ population described in this study—which was present in two of the more prevalent clusters included in the described Vδ2^−^ signature—may help to more accurately discern targetable immune populations in immuno-oncology. Finally, our study highlights their capacity to endure more adverse milieus than their αβ T-cell counterparts. Currently, Vδ1 T cells are the only Vδ2^−^ cells for which selective expansion and clinical application methods have been optimized. Developing treatments that more specifically target any activatable γδ T-cell subsets could enable more potent cancer immunotherapies.

## Methods

### Human samples

Tumor samples were obtained from resection surgery from adult patients with histologically confirmed RCC. The institutional review boards of all participating institutions approved the study protocol and studies were performed in accordance with the US Common Rule. All patients gave written informed consent before participation in the study.

Healthy tissues were obtained from deceased organ donors as part of organ acquisition for clinical transplantation through an approved protocol and material transfer agreement with LiveOnNY as described previously^73^. Donors were previously healthy and free of cancer and chronic diseases, and seronegative for hepatitis B and C and human immunodeficiency virus. This study does not qualify as ‘human subjects’ research, as confirmed by the Columbia University institutional review board as tissue samples were obtained from brain-dead (deceased) individuals of both biological genders, of white, Black/African American, Hispanic and Asian ethnicity, with ages ranging from 23 to 92 years old.

### Tissue sample preparation

Surgical specimens were obtained fresh from the operating field, and dissected in surgical pathology, where tumors were isolated, minced and transported at room temperature (RT) immersed in L15 medium with 15 mM HEPES and 600 mg ml^−1^ glucose. Resected tumor kidney tissues were further minced using surgical scissors in GentleMACs C Tubes (Miltenyi Biotec) containing RPMI-1640 medium with 0.1 mg ml^−1^ of Liberase TL and 0.2 mg ml^−1^ of DNase I. Samples were then simultaneously mechanically and enzymatically digested for 1 h at 37 °C using a GentleMACS dissociator (Miltenyi Biotec), according to the manufacturer TDK-1 tumor protocol. The digestion reaction was quenched with FACS buffer (phosphate-buffered saline (PBS) buffer supplemented with 2% heat-inactivated fetal calf serum (FCS) and 2 mM EDTA) and filtered through a 100 μm cell strainer. Samples were resuspended in a 155 mM NH_4_Cl solution to lyse all erythrocytes and washed in FACS buffer. Finally, the samples were either frozen for storage or resuspended for flow cytometry staining or scRNA-seq. Peripheral blood mononuclear cells (PBMCs) were obtained by Ficoll-gradient centrifugation. Tissues from healthy individuals were maintained in cold saline or University of Wisconsin solution and transported to the laboratory within 8–10 h of organ procurement. Lymphocytes were isolated from blood and bone marrow samples by density centrifugation using lymphocyte separation medium (Corning, cat. no. 25-072-CI) for recovery of mononuclear cells. Spleen, lung, intestinal, tonsil and LN samples were processed using enzymatic and mechanical digestion, resulting in high yields of live leukocytes, as previously described^73,74^. Data collection and analysis were not performed blind to the conditions of the experiments. Randomization was not relevant for the samples analyzed in this study.

### Cell culture and cell stimulation

Frozen aliquots of single-cell suspension from tissues were thawed and maintained in RPMI-1640 medium supplemented with 10% heat-inactivated FCS, 1 mM glutamine, 1 mM sodium pyruvate, 1 mM non-essential amino acids, 1% penicllin–streptomycin and 0.2 mg ml^−1^ DNase I. For intracellular and cytokine staining, cells were stimulated with 1× cell stimulation cocktail plus protein transport inhibitors (eBioscience) and incubated for 3–4 h at 37 °C. Cells were then washed in PBS to remove culture medium to proceed for flow cytometry staining.

### Flow cytometry

Conventional or high-parameter surface flow cytometry staining was performed in 15 ml conical or V-bottomed 96-well plates. Briefly, fresh or thawed frozen tissues from up to 22 RCC and healthy donors were processed to single-cell suspension were washed with PBS, resuspended in 1 ml of viability dye and incubated at RT in the dark for 10 min for FVS575V or 30 min for Live/Dead Fixable Aqua. Following, samples were washed once with cold FACS buffer and resuspended with the first antibody mix composed of anti-γδ TCR antibody, human TrueStain FcX and mouse serum. After incubation on ice for 10 min, the rest of the antibodies (Reporting Summary) were added together with 50 μl of Horizon Brilliant Stain buffer and incubated for an additional 20 min on ice. After incubation, cells were washed twice with FACS buffer and resuspended in FACS buffer for same-day acquisition or fixed in 100 μl FluoroFix on ice for 20 min and washed once for following-day analysis. For cytokines and intracellular staining, cells were additionally fixed for 30 min at RT with 100 μl of Foxp3/Transcription Factor Staining Buffer Set (eBioscience). After incubation, cells were washed once in permeabilization buffer followed by resuspension in the antibody mix. After incubation for 30 min at RT, cells were washed with FACS buffer and resuspended in FACS buffer for analysis.

Samples were analyzed on an LSR Fortessa X50 cytometer or FACSAria Fusion (BD Bioscience). Instrument day-to-day variability was adjusted for using BD FACSDiva CS&T Research Beads or Rainbow Calibration particles. Mean fluorescence intensity (MFI) of the fluorescent beads was recorded for each channel on the first day of acquisition of the first samples. On following acquisition days, laser voltages were adjusted to match the MFI intensity acquired on the first day. After voltage adjustment, instrument compensation was recorded each day before sample acquisition using single staining bead controls (ArC reactive beads, UltraComp eBeads or OneComp Beads). Data were analyzed with FlowJo 10. For multi-dimensional analyses, γδ^+^ T-cell populations and Vδ2^+^ and Vδ2^−^ subpopulations (defined as live, CD45^+^CD14^−^CD19^−^CD3^+^ γδ^−^TCR^+^ cells and further divided according to Vδ2 expression) were exported and analyzed in R using Grappolo, Vite and Panorama algorithms by an established workflow (https://github.com/ParkerICI/flow-analysis-tutorial and https://github.com/ParkerICI). Force-directed graphs were arranged using the graphic software Gephi.

### Flow cytometry cell sorting

Samples from six patients with RCC were used for transcriptome and TCR-sequencing analyses. Six healthy donor peripheral blood samples were used as controls. Samples were stained according to the protocol described above. All samples were sorted by flow cytometry (FACSAria Fusion, BD Bioscience) for Vδ2^−^γδ^+^ and Vδ2^+^γδ^+^ T cells.

### Statistical analysis

Graphs and statistical analyses were generated and calculated using GraphPad Prism. After testing for normality tests, the comparison of the surface expression of markers across multiple cell populations was calculated using the Friedman test for matched nonparametric multiple comparisons. To compare the frequency of two populations or the expression of markers between two groups, the Wilcoxon test for paired nonparametric comparisons was used. For comparing the populations frequencies between different tissues, statistics were calculated by one-way analysis of variance (ANOVA) on log_10_-transformed data to account for differential distribution of the populations between different tissues. Differential distribution was tested using the Brown–Forsythe and the Barlett’s tests. Statistical tests run with clinical trial datasets are described in the ‘Expression analysis’ section in Methods. No data were excluded from analyses.

### RNA sequencing

Tumor samples from six patients with RCC were digested in RPMI-1640 containing collagenase I and II (0.1 mg ml^−1^, Sigma-Aldrich) and DNAse I, minced, and digested for 1 h using the GentleMACS system (Miltenyi Biotec). Live γδ, CD4 and CD8 T cells were isolated from each digest using the BD FACSAria Fusion flow cytometer. Bulk RNA libraries (used for demultiplexing) were prepared according to the Smart-seq2 protocol^75^ and sequenced on an Illumina HiSeq 4000. Samples were then pooled by cell type (into γδ, CD4 and CD8 T-cell groups) before library preparation. Libraries were constructed using 10x Genomics Chromium 5′ (v1) and 10x Genomics Chromium V(D)J kit (PN-1000005) and sequenced on an Illumina NovaSeq 6000 with paired-end 100 base pair read lengths. For γδ TCR amplification, custom oligonucleotide sequences displayed in Supplementary Table 2 were used.

### Expression analysis

Alignment and assembly of raw scRNA-seq data were done with CellRanger version 3.1.0 (10x Genomics, Genome Build: GRCh38 3.0.0). In finalizing the alignment and assembly of our γδ TCR-enriched library, to address a lack of full support for gamma delta V(D)J contig annotation in v3.1.0, we used a custom script to process the original annotated contig outputs (from Cell Ranger) for productive contigs as defined by the six conditions listed at https://support.10xgenomics.com/single-cell-vdj/software/pipelines/3.1/algorithms/annotation. For further preprocessing and analysis, we used the Scanpy^76^ single-cell analysis toolkit (scanpy 1.7.2, pandas 1.2.4 and numpy 1.20.2).

Upon loading the data from the γδ sort, Demuxlet^77^ was used for doublet removal and demultiplexing our sorted sample pools into six patient samples. Cells with fewer than 200 genes expressed and genes detected in fewer than three cells were excluded. For further quality control, we used only cells with <4,000 genes, <20,000 counts and <10% mitochondrial genes. Reads were normalized to 10,000 per cell, and a log + 1 transformation was applied. For visualization only, genes were subset to highly variable genes using the default Scanpy preprocessing function, counts and percent mitochondrial genes were regressed out, and data were scaled to unit variance and zero mean. The dimensionality reduction (for 2D visualization) was calculated using uniform manifold approximation and projection (UMAP) algorithm. Clustering was performed using the Leiden algorithm (res = 0.6, leidenalg 0.8.4). Vδ2^+/−^ labels were assigned according to the most strongly expressed Vδ gene. Gene set scores were calculated using Scanpy’s ‘scanpy.tl.score_genes’ function.

For the Vδ2^−^ gene signature analysis, pooled data from the γδ, CD4 and CD8 T-cell sorts were used. We prepared our data in the same manner as with the γδ-only analysis (that is demultiplexing with Demuxlet, the same preprocessing settings and macrophage removal), with an additional batch correction of samples using Scanpy’s ‘scanpy.pp.combat’ function. To generate the Vδ2^−^ signature, Vδ2^−^ γδ T cells were compared against Vδ2^+^ γδ T cells, CD4 αβ T cells and CD8 αβ T cells in a differential expression analysis using the Wilcoxon rank-sum test, and the top 20 most upregulated genes by *P* value were selected. Volcano plots were produced using the bioinfokit toolkit (bioinfokit 2.0.6). For the Kaplan–Meier survival curve analysis, xCell cell type enrichment analysis^78^ was used to assign Vδ2^−^ signature scores to bulk RNA data from TCGA RCC samples. xCell Vδ2^−^ signature scores were also calculated for bulk RNA samples (grouped by clinical response, Wilcoxon rank-sum test) from the IMmotion150 (RCC) and IMvigor210 (urothelial) anti-PD-L1 checkpoint inhibitor trials in their respective clinical response analyses.

### γδ T-cell clonal expansion, TCR repertoire diversity and social network analysis

To annotate our scRNA data with data from our γδ TCR-enriched library for clonal expansion analysis, we read in our processed annotated contig file, selecting only productive contigs for further use. Contigs with TRAV_DV_ V genes (for example*, TRAV29DV5*) were labeled as delta contigs if the D, J and C genes were also delta genes. A table of paired γδ TCRs was generated by merging gamma and delta contig rows with shared cell barcodes. For each sample, paired TCR sequences were defined by concatenating gamma and delta nucleotide sequences. Cells were then labeled as ‘expanded’ when more than one cell within the sample shared the same sequence and as ‘singleton’ when no other cell within the sample shared the same sequence.

The TCR social network analysis was done at cell level. Cells without phenotypic cluster numbers obtained from analysis of the scRNA-seq data were excluded from the network analysis. For each patient, pairwise Levenshtein distances between delta-chain CDR3 amino acid sequences were calculated to obtain the distance matrix (R package: NAIR, https://github.com/mlizhangx/Network-Analysis-for-Repertoire-Sequencing-). Only TCRs with same amino acid sequence in their delta chain were connected (distance = 0). The network analysis was plotted using R packages igraph and ggraph^79^. The number of clusters belonging to each network and the maximum cluster size (the maximum number of clones belonging to each cluster across all clusters within the network) were used to describe the network properties. Both values were normalized by the number of cells and log_10_ transformed. Gini coefficients were used to quantitate the diversity of the TCR repertoire and calculated on the basis of the number of cells per patient belonging to a single delta-chain clone within the TCR repertoire. Statistical comparisons of Gini coefficients across groups were performed using Wilcoxon signed-rank test.

### Autologous tumor killing assays

γδ^+^ Vδ2^+^, γδ^+^ Vδ2^−^ or αβ CD8^+^ cells were sorted from a vial of previously digested and frozen kidney tumor using a FACSAria Fusion (Becton Dickinson). CD8 T cells were activated with Dynabeads human T-activator CD3/CD28/CD137 (Gibco by Life Technologies) at 1:5 beads-to-T-cell ratio, and γδ^+^ cells were activated with gamma-irradiated autologous tumor at 2:1 tumor-to-γδ-cell ratio. T cells were cultured in ImmunoCult-XF T cell expansion medium (StemCell Technologies) with 10% human AB serum (heat inactivated), 100 U ml^−1^ penicillin and streptomycin, 50 µg ml^−1^ gentamicin, 200 U ml^−1^ IL-2 (Peprotech) and 10 ng ml^−1^ each of IL-7, IL-15 and IL-21 (Peprotech) in a well of a U-bottom 96-well plate and incubated at 37 °C, 5% CO_2_ and 95% relative humidity. Half of media was changed weekly, and cells were expanded into two wells only when medium was orange-yellow. With each expansion, IL-2 concentration was increased from 200 U ml^−1^ to 500 U ml^−1^, and then to 1,000 U ml^−1^ and, if needed, to 2,000 U ml^−1^ until sufficient T cells were obtained. Autologous tumor cells were sorted (live CD45^−^CD3^−^) from another aliquot of frozen tumor cells from the same patient, and 3,000–5,000 cells were plated per well in complete media (RPMI-1640, 1% non-essential amino acids, 1 mM sodium pyruvate, 2 mM l-glutamine, 100 U ml^−1^ penicillin–streptomycin and 10% heat-inactivated FBS) in a flat-bottom 96-well plate and incubated at 37 °C till overnight. Anti-PD-L1 antibody (atezolizumab) was added to tumor cells at a final concentration of 10 μg ml^−1^ and incubated at RT for 30 min. Dead T cells were removed from expanded T cells using the EasySep Dead Cell Removal (Annexin V) Kit (StemCell Technologies) before plating live T cells with tumor cells in a ratio of 40:1 and Annexin V Green reagent (Essen Bioscience, 1 μl per 200 ml of medium) was added. Cells were monitored by time-lapse microscopy using a IncuCyte Zoom system (Essen Bioscience) at 1 h intervals. Analysis was performed using the IncuCyte Zoom software. All traces are displayed as relative change in cell death from timepoint 0 with background death of T cells at each timepoint subtracted (as average of triplicates).

### ELISpot from lymphocyte–tumor co-cultures

γδ Vδ2^+^, Vδ2^−^, αβ CD8 T cells and EpCAM^+^ kidney tumor cells (BioLegend 324224, clone 9C4) were sorted from freshly digested tumors. Six hundred T cells and 300 tumor cells were co-incubated in anti-IFNγ antibody (Mabtech 3420-2A)-coated ELISpot plates (Mabtech 3654-WP-10) for 48 h in a 37 °C CO_2_ incubator. Medium used was RPMI containing heat-inactivated 10% human AB serum, 1% non-essential amino acids, 1 mM sodium pyruvate, 2 nM l-glutamine, 100 U ml^−1^ penicillin–streptomycin, 50 µg ml^−1^ of gentamicin, 200 U of IL-2 and 10 ng ml^−1^ each of IL-7, IL-15 and IL-21 (Peprotech). IFNγ-positive spots were detected with biotinylated detection antibody, streptavidin-ALP and BCIP/NBT-plus (Mabtech, 3650-10) according to the manufacturer’s instructions. Quantitation was carried out using the CTL Immunospot 5.0 Analyzer. Statistical analysis was performed by one-way ANOVA with nonparametric settings (Kruskal–Wallis test) in GraphPad Prism.

### Reporting summary

Further information on research design is available in the Nature Portfolio Reporting Summary linked to this article.

## Online content

Any methods, additional references, Nature Portfolio reporting summaries, source data, extended data, supplementary information, acknowledgements, peer review information; details of author contributions and competing interests; and statements of data and code availability are available at 10.1038/s41590-023-01448-7.

## Supplementary information

Reporting Summary

Supplementary Table 1 Gene expression analysis to generate the Vδ2^−^ specific signature.

Supplementary Table 2 Custom oligonucleotides used for γδ TCR amplification.

## Data Availability

Sequencing data have been deposited in GEO under the accession code GSE223809. Clinical datasets used to interrogate gene signatures are from NCT01984242 and NCT02108652 clinical trials. All other data are available in the article and Supplementary Information or from the corresponding author upon reasonable request.

## Code availability

Code used to analyze the RNA sequencing data in this manuscript is available at https://github.com/fonglab/pub-gd. All other code related to this study is available from the corresponding author on reasonable request.
