# Supplementary material for: Exhausted intratumoral Vδ2− γδ T cells in human kidney cancer retain effector function
Source: Nat Immunol. 2023 Mar 16;24(4):612–24. doi: 10.1038/s41590-023-01448-7 (PMC10063448; doi:10.1038/s41590-023-01448-7)
Supplement: Supplementary file 1 — Reporting Summary [file 41590_2023_1448_MOESM1_ESM.pdf]

nature portfolio

Corresponding author(s): Lawrence Fong

Last updated by author(s): Jan 9, 2023

Reporting Summary

Nature Portfolio wishes to improve the reproducibility of the work that we publish. This form provides structure for consistency and transparency in reporting. For further information on Nature Portfolio policies, see our Editorial Policies and the Editorial Policy Checklist.

Statistics

For all statistical analyses, confirm that the following items are present in the figure legend, table legend, main text, or Methods section.

n/a	Confirmed	
☐	☒	The exact sample size (n) for each experimental group/condition, given as a discrete number and unit of measurement
☐	☒	A statement on whether measurements were taken from distinct samples or whether the same sample was measured repeatedly
☐	☒	The statistical test(s) used AND whether they are one- or two-sided<br>*Only common tests should be described solely by name; describe more complex techniques in the Methods section.*
☐	☒	A description of all covariates tested
☐	☒	A description of any assumptions or corrections, such as tests of normality and adjustment for multiple comparisons
☐	☒	A full description of the statistical parameters including central tendency (e.g. means) or other basic estimates (e.g. regression coefficient) AND variation (e.g. standard deviation) or associated estimates of uncertainty (e.g. confidence intervals)
☐	☒	For null hypothesis testing, the test statistic (e.g. F, t, r) with confidence intervals, effect sizes, degrees of freedom and P value noted<br>*Give P values as exact values whenever suitable.*
☒	☐	For Bayesian analysis, information on the choice of priors and Markov chain Monte Carlo settings
☒	☐	For hierarchical and complex designs, identification of the appropriate level for tests and full reporting of outcomes
☒	☐	Estimates of effect sizes (e.g. Cohen's d, Pearson's r), indicating how they were calculated

Our web collection on statistics for biologists contains articles on many of the points above.

Software and code

Policy information about availability of computer code

Data collection	Flow cytometry: FACSDiva 9.0; Sequencing data alignment and assembly: CellRanger 3.1.0; pre-processing: Scanpy toolkit (scanpy 1.7.2, pandas 1.2.4, numpy 1.20.2); Time-lapse microscopy: IncuCyte Zoom System 2021A
Data analysis	Flow cytometry: FlowJo 10, Grappolo Vite and Panorama (R based); Sequencing data Demultiplexing: Gephi 0.9, Demuxlet; Analysis: Scanpy toolkit (scanpy 1.7.2, pandas 1.2.4, numpy 1.20.2); TCR analysis: Scanpy 1.7.2, NAIR 0.0.9009, igraph, ggraph (R based); Kaplan-Meier and clinical response analyses: xCell enrichment; Time-lapse microscopy pre-processing: IncuCyte integrated analysis 2021A

For manuscripts utilizing custom algorithms or software that are central to the research but not yet described in published literature, software must be made available to editors and reviewers. We strongly encourage code deposition in a community repository (e.g. GitHub). See the Nature Portfolio guidelines for submitting code & software for further information.

Data

Policy information about availability of data

All manuscripts must include a data availability statement. This statement should provide the following information, where applicable:

- Accession codes, unique identifiers, or web links for publicly available datasets
- A description of any restrictions on data availability
- For clinical datasets or third party data, please ensure that the statement adheres to our policy

The datasets generated during and/or analysed during the current study are available from the corresponding author on reasonable request.

Field-specific reporting

Please select the one below that is the best fit for your research. If you are not sure, read the appropriate sections before making your selection.

☒ Life sciences ☐ Behavioural & social sciences ☐ Ecological, evolutionary & environmental sciences

For a reference copy of the document with all sections, see nature.com/documents/nr-reporting-summary-flat.pdf

Life sciences study design

All studies must disclose on these points even when the disclosure is negative.

Sample size	Sample size was restricted by availability of human specimens.
Data exclusions	No data were excluded.
Replication	Triplicates measurements were performed in the in vitro stimulation experiments. Due to the perishable nature of patient specimens, this was not possible with experiments using primary cells.
Randomization	Minimization of individual or batch effects was pursued in sample submission for RNA sequencing by pooling samples from different donors/ patients in the same reaction. No experimental group randomization was applied for renal cell carcinoma immunophenotyping. For in vitro experiments, expanded or non-expanded lymphocyte were allocated to each experimental condition from the same cell container.
Blinding	Sample staining and sequencing were performed in batches that contain the different samples and/or experimental conditions. Acquisition of the data (e.g. sequencing) was blinded to the patient group, and deconvoluted with the analyses. Blinding was not applied for biopsy processing and analysis. For in vitro studies, blinding was not possible since the operators who performed the experiments were the same that analyzed them.

Reporting for specific materials, systems and methods

We require information from authors about some types of materials, experimental systems and methods used in many studies. Here, indicate whether each material, system or method listed is relevant to your study. If you are not sure if a list item applies to your research, read the appropriate section before selecting a response.

Materials & experimental systems

n/a	Involved in the study	
☐	☒	Antibodies
☒	☐	Eukaryotic cell lines
☒	☐	Palaeontology and archaeology
☒	☐	Animals and other organisms
☐	☒	Human research participants
☒	☐	Clinical data
☒	☐	Dual use research of concern

Methods

n/a	Involved in the study	
☒	☐	ChIP-seq
☐	☒	Flow cytometry
☒	☐	MRI-based neuroimaging

Antibodies

Antibodies used	LiveDead Aqua from Invitrogen (L34957) was used for viability. The following anti-human antibodies were used for flow cytometry analysis and/or sorting. From Biolegend: 302046 (CD16, clone 3G8), 302246 (CD19, clone HIB19), 328230 (CD39, clone A1), 318334 (CD56, clone HCD56), 300920 (CD8, clone HIT8a), 369616 (CTLA4, clone BNI3), 320125 (FoxP3, clone 206D), 331210 (γδ TCR, clone B1), 502312 (GM-CSF, clone BVD2-21C11), 313536 (ICOS, clone C398.4A), 501413 (IL-10, clone JES3-9D7), 512323 (IL-17A, clone BL168), 500350 (IL-2, clone MQ1-17H12), 501118 (IL-6, clone MQ2-13A5), 514606 (IL-8, clone BH0814), 350520 (Ki67, clone ki67), 329920 (PD-1, clone EH12.2H7), 353320 (Perforin, clone B-D48), 372714 (TIGIT, clone A15153G), 345028 (TIM3, clone F38-2E2), 502915 (TNFalpha, clone MAb11). From BD Biosciences: 741000 (4-1BB, clone 4B4-1), 565779 (CD14, clone M5E2), 564001 (CD3, clone SK7), 555348 (CD4, clone RPA-T4), 566355 (CD4, clone SK3), 564585 or 564915 (CD45, clone HI30), 564804 (CD8, clone RPA-T8), 564620 (IFNgamma, clone 4S.B3). From Miltenyi Biotech: 130-095-803 (Vδ2 TCR, clone 123R3).<br><br>Functional anti human PD-L1 (Atezolizumab, Tecentriq) was used in ex vivo stimulation experiments.
Validation	Concentration titrations and FMOs were performed for all antibodies used. In detail, PBMCs were stained with a range of concentrations for each antibody and analyzed for optimal staining, using manufacturer's data as reference to determine final dilutions. For multicolor flow cytometry experiments, Fluorescence-Minus-One (FMO) tubes were prepared for each marker by staining PBMCs with the full staining cocktail except the antibody for the relevant marker. This validation was not feasible with biopsy RCC samples due to the limited amount of specimen.

Human research participants

Policy information about studies involving human research participants

Population characteristics	Tissues from individuals without cancer were obtained from deceased organ donors as part of organ acquisition for clinical transplantation through an approved protocol and material transfer agreement with LiveOnNY as described previously (https://www.sciencedirect.com/science/article/pii/S0092867420301033?via%3Dihub). Donors were free of cancer, chronic diseases, seronegative for hepatitis B, C, and HIV, and represented diverse ages and both sexes. (https://www.sciencedirect.com/science/article/pii/S0092867420301033?via%3Dihub). Cancer and normal tissues were also obtained from patients undergoing resection for kidney tumors. Patients represented both biological genders, of White, black/African American, Hispanic and Asian ethnicity, with ages ranging 23 to 92 years old. .
Recruitment	Tissues from individuals without cancer were obtained from deceased organ donors as part of organ acquisition for clinical transplantation the LiveOnNY. Cancer patients were undergoing surgery for kidney tumors at UCSF. . All patients gave written informed consent prior to participation in the study.
Ethics oversight	The study on non-cancer human tissues does not qualify as "human subjects" research, as confirmed by the Columbia University Institutional Review Board (IRB) as tissue samples were obtained from brain-dead (deceased) individuals. The study on kidney cancer tissues was approved by the UCSF Human Research Proteciton Program.

Note that full information on the approval of the study protocol must also be provided in the manuscript.

Flow Cytometry

Plots

Confirm that:

☒ The axis labels state the marker and fluorochrome used (e.g. CD4-FITC).

☒ The axis scales are clearly visible. Include numbers along axes only for bottom left plot of group (a 'group' is an analysis of identical markers).

☒ All plots are contour plots with outliers or pseudocolor plots.

☒ A numerical value for number of cells or percentage (with statistics) is provided.

Methodology

Sample preparation	Samples were brought to single cell suspension, then washed with PBS, resuspended in 1 mL of viability dye and incubated at room temperature in the dark for 10 minutes for FVS575V or 30 minutes for Live/Dead Fixable Aqua. Following, samples were washed once with cold FACS-buffer and resuspended with the first antibody mix comprised of the anti-γδ TCR antibody, human TrueStain FcX, and mouse serum. After incubation on ice for 10 minutes, the rest of the antibodies were added together with 50 µl of Horizon Brilliant Stain buffer and incubated for an additional 20 minutes on ice. After incubation, cells were washed twice with FACS-buffer and resuspended in FACS-buffer for same-day acquisition or fixed in 100 µl FluoroFix on ice for 20 minutes and washed once for following day analysis. For cytokines and intracellular staining, cells were additionally fixed for 30 minutes at RT with 100 µl of Foxp3/Transcription factor Staining Buffer Set (eBioscience). After incubation, cells were washed once in permeabilization buffer followed by resuspension in the antibody mix. After incubation for 30 minutes at RT, cells were washed with FACS-buffer and resuspended in FACS-buffer for analysis.
Instrument	LSR Fortessa X50 and FACSAria Fusion
Software	Collection: FACSDiva; Analysis: FlowJo 10, Grappolo Vite, Panorama
Cell population abundance	Within RCC tumor samples, ~60% CD45+CD3+ were found and further sorted into αβ or γδ subsets. In average, 3-7% of those were sorted as Vδ2-, while 0.5-2% as Vδ2+.
Gating strategy	For immunophenotyping, singles cells were manually gated in sequential Time/SSC-A (low Time low SSC-A), FSC-A/SSC-A (leaving S-low/F-low events out), FSC-A/FSC-W (taking only events in the diagonal correlating both signals) and SSC-A/SSC-W (same as previous). LiveDead-CD45+ events were then gated and further analyzed for marker expression.<br>For sorted samples, singlet gating was performed by sequential FSC-A/FSC-W and SSC-A/SSC-W manual gating of proportional events (in diagonal). LiveDead- SSC-A was used to isolate viable cells, which were further gated based on CD45/CD3 expression.<br>In all cases, unstained and/or FMO samples were used as reference for positive staining. Single-stained compensation controls were included in all runs.

☒ Tick this box to confirm that a figure exemplifying the gating strategy is provided in the Supplementary Information.

March 2021
