# Supplementary material for: Exhausted intratumoral Vδ2− γδ T cells in human kidney cancer retain effector function
Source: Nat Immunol. 2023 Mar 16;24(4):612–24. doi: 10.1038/s41590-023-01448-7 (PMC10063448; doi:10.1038/s41590-023-01448-7)
Supplement: Supplementary file 2 — Gene expression analysis to generate the Vδ2− specific signature. [file 41590_2023_1448_MOESM2_ESM.pdf]

**Supplementary Table 1. Gene expression analysis to generate the Vδ2- specific signature**

| Vd2+_name | Vd2+_pval | Vd2+_logf | Vd2-_name | Vd2-_pval | Vd2-_logf | nan_name | nan_pval | nan_logf |
|---|---|---|---|---|---|---|---|---|
| TRDV2 | 0 | 8.874661 | TRDV1 | 0 | 6.431989 | CD8A | 8.85E-223 | 2.725935 |
| TRGV9 | 1.32E-259 | 4.724355 | TRDV3 | 3.44E-10 | 5.594638 | CD8B | 1.44E-209 | 2.986982 |
| RPL41 | 4.35E-147 | 0.727017 | TRDC | 5.24E-213 | 4.312954 | SIT1 | 1.38E-110 | 1.703556 |
| RPL36A | 3.83E-104 | 0.984643 | KIR3DL2 | 2.38E-16 | 3.894518 | LAPTM5 | 6.38E-107 | 0.584622 |
| TRDC | 3.55E-103 | 3.652212 | KIR2DL3 | 2.50E-14 | 3.745843 | CD2 | 4.02E-95 | 0.738666 |
| KLRD1 | 6.50E-100 | 2.577066 | TRGV4 | 3.03E-37 | 3.715089 | LTB | 1.08E-76 | 1.146078 |
| RPS10 | 4.06E-97 | 0.946513 | KLRC2 | 1.62E-23 | 3.188574 | ICOS | 1.19E-74 | 1.723144 |
| RPS29 | 3.82E-80 | 0.584972 | TYROBP | 3.02E-21 | 3.013426 | CORO1B | 2.43E-74 | 1.264751 |
| GNLY | 5.75E-72 | 3.009347 | KLRC3 | 5.89E-80 | 3.002368 | CD4 | 2.43E-64 | 4.765623 |
| MATK | 1.75E-67 | 1.846779 | AC068775.1 | 2.16E-17 | 2.934163 | CD27 | 8.15E-62 | 0.917356 |
| KLRG1 | 1.80E-59 | 1.899582 | TRGV8 | 2.70E-21 | 2.805238 | BATF | 8.84E-58 | 1.152697 |
| ZNF683 | 2.51E-51 | 2.108055 | SYNGR1 | 6.65E-08 | 2.804379 | SOD1 | 1.55E-55 | 0.551434 |
| HOPX | 9.67E-48 | 1.365078 | LYN | 5.47E-11 | 2.692133 | B2M | 5.49E-51 | 0.189482 |
| CTSW | 6.01E-46 | 1.142051 | TRGC2 | 1.20E-41 | 2.586837 | SPOCK2 | 2.37E-49 | 0.75567 |
| GZMM | 1.31E-44 | 1.156084 | KLRD1 | 1.67E-164 | 2.516608 | DNAJB1 | 1.87E-48 | 0.887734 |
| GZMB | 2.44E-43 | 1.768394 | LAT2 | 1.24E-29 | 2.514401 | CTSB | 2.10E-45 | 1.332619 |
| LINC01871 | 1.94E-42 | 1.544522 | CD160 | 2.73E-08 | 2.422441 | CACYBP | 1.56E-43 | 0.680271 |
| TRG-AS1 | 2.13E-40 | 1.813174 | TRGC1 | 5.30E-07 | 2.370853 | IL7R | 5.00E-38 | 1.030699 |
| LYAR | 2.12E-39 | 1.582288 | TRGV5 | 3.44E-11 | 2.369953 | CD82 | 6.67E-38 | 0.800996 |
| NKG7 | 8.11E-38 | 1.471588 | LINC02446 | 6.32E-97 | 2.367771 | LAT | 4.87E-37 | 0.644786 |
| XCL2 | 2.89E-37 | 1.824595 | ZNF683 | 1.37E-119 | 2.355994 | CD5 | 5.87E-36 | 1.050933 |
| TRBC1 | 8.95E-35 | 1.047565 | TRGV3 | 5.92E-28 | 2.22718 | HSPA1A | 1.28E-35 | 0.882493 |

| | | | | | | | | |
|---|---|---|---|---|---|---|---|---|
| CCL4 | 4.98E-33 | 1.263464 | XCL2 | 4.64E-115 | 2.191258 | HNRNPLL | 1.82E-35 | 0.703974 |
| CCL5 | 2.25E-32 | 1.307414 | TRGV7 | 1.82E-12 | 2.110046 | CD3E | 2.74E-35 | 0.257215 |
| GZMH | 1.60E-30 | 1.145806 | PLCG2 | 9.50E-11 | 2.070577 | ITM2B | 6.14E-34 | 0.336498 |
| KLRC1 | 5.52E-30 | 3.004067 | FCRL6 | 7.59E-34 | 1.935575 | HSPA1B | 2.81E-31 | 0.771001 |
| HCST | 9.18E-30 | 0.644079 | NCR3 | 7.80E-40 | 1.865198 | TNFRSF4 | 4.76E-31 | 2.461088 |
| KLRB1 | 4.84E-26 | 1.309723 | AOAH | 6.14E-71 | 1.831436 | CD28 | 2.19E-29 | 1.512486 |
| LINC02446 | 7.49E-25 | 1.610256 | TRGV2 | 8.08E-16 | 1.831169 | RPL12 | 9.95E-29 | 0.17642 |
| XCL1 | 5.29E-24 | 1.437491 | XCL1 | 2.98E-79 | 1.801894 | CTLA4 | 1.23E-28 | 1.301258 |
| RPS27 | 5.74E-24 | 0.242253 | FAM49A | 2.21E-10 | 1.745354 | HLA-B | 6.39E-28 | 0.199336 |
| ANXA1 | 6.00E-23 | 0.89017 | SPRY2 | 4.01E-08 | 1.741605 | HSP90AA1 | 3.29E-27 | 0.477423 |
| ITM2C | 1.09E-20 | 0.855696 | MATK | 1.14E-106 | 1.739982 | HSPB1 | 9.34E-27 | 0.525744 |
| NCR3 | 5.29E-19 | 1.724147 | SERPINB6 | 1.91E-07 | 1.682888 | CD40LG | 4.76E-26 | 2.467778 |
| GABARAPL1 | 5.37E-18 | 0.999513 | TRGV9 | 3.27E-20 | 1.647731 | HSP90AB1 | 8.83E-26 | 0.424863 |
| RPL23A | 8.73E-18 | 0.330424 | KLRK1 | 2.97E-13 | 1.623166 | HINT1 | 3.95E-25 | 0.287101 |
| CD52 | 2.70E-17 | 0.572484 | IFITM3 | 3.49E-13 | 1.532527 | BAG3 | 9.16E-24 | 0.673645 |
| VIM | 3.76E-17 | 0.55238 | TRG-AS1 | 9.28E-51 | 1.532145 | PBXIP1 | 4.83E-23 | 0.76821 |
| IFITM1 | 2.54E-15 | 0.579489 | CTSW | 4.50E-185 | 1.508273 | S100A4 | 5.86E-23 | 0.361046 |
| TYROBP | 2.65E-15 | 2.865456 | IKZF2 | 2.47E-34 | 1.507252 | TOX2 | 9.94E-23 | 1.720831 |
| RPS3 | 3.05E-15 | 0.210564 | APBA2 | 1.59E-06 | 1.479429 | ANAPC16 | 1.80E-22 | 0.384796 |
| FKBP11 | 4.13E-15 | 1.019466 | CRTAM | 1.51E-45 | 1.464828 | LDHB | 3.48E-22 | 0.330397 |
| MT2A | 7.28E-15 | 0.719373 | C1orf21 | 1.27E-07 | 1.445609 | C12orf57 | 6.10E-22 | 0.332646 |
| RPS12 | 1.44E-13 | 0.221727 | ERGIC1 | 7.17E-06 | 1.40477 | FYB1 | 9.01E-22 | 0.386485 |
| REL | 1.86E-13 | 0.791654 | CCL5 | 4.00E-117 | 1.394404 | PAG1 | 1.87E-21 | 0.899957 |
| TRAC | 2.46E-13 | 0.724783 | ZEB2 | 1.58E-30 | 1.378962 | FKBP1A | 2.42E-21 | 0.46997 |

| | | | | | | | | |
|---|---|---|---|---|---|---|---|---|
| ALOX5AP | 3.49E-13 | 0.669848 | GNLY | 5.70E-22 | 1.372183 | UBC | 2.76E-20 | 0.294624 |
| RPS21 | 4.71E-13 | 0.218326 | PTGDR | 2.45E-09 | 1.359516 | AQP3 | 9.14E-20 | 1.273257 |
| CLIC3 | 4.91E-13 | 1.117385 | ABCB1 | 1.75E-11 | 1.326556 | HSPA8 | 2.62E-19 | 0.332451 |
| TRGC1 | 5.41E-13 | 3.302433 | KLRC4 | 1.52E-13 | 1.296002 | FXYD5 | 3.83E-19 | 0.260682 |
| RPL37A | 1.55E-12 | 0.351423 | PIK3AP1 | 4.58E-13 | 1.291423 | TMEM173 | 4.18E-19 | 0.989282 |
| NOSIP | 2.37E-12 | 0.834279 | YBX3 | 4.28E-08 | 1.273787 | PEBP1 | 5.35E-19 | 0.406017 |
| RPS2 | 4.08E-12 | 0.241837 | DGKD | 5.58E-08 | 1.233094 | DNAJB4 | 8.51E-19 | 0.719783 |
| ADRB2 | 6.48E-12 | 1.480309 | KRT86 | 2.13E-06 | 1.212583 | SYNGR2 | 4.52E-18 | 0.553507 |
| HIST2H2AA4 | 7.13E-12 | 0.728554 | NKG7 | 1.35E-57 | 1.193127 | S100A11 | 2.17E-17 | 0.381763 |
| MBP | 7.80E-12 | 0.755427 | METRNL | 1.07E-06 | 1.183404 | ARL6IP5 | 7.48E-17 | 0.30211 |
| S100A10 | 2.60E-11 | 0.494977 | TBCD | 1.11E-17 | 1.142459 | PDCD1 | 9.28E-17 | 0.621078 |
| CD247 | 2.75E-11 | 0.541372 | CD244 | 1.54E-07 | 1.13684 | SIRPG | 1.31E-16 | 0.627798 |
| UPP1 | 3.27E-11 | 0.805601 | RRAS2 | 7.57E-06 | 1.076066 | TMBIM6 | 2.01E-16 | 0.246723 |
| SEMA4D | 5.68E-11 | 0.725832 | GZMB | 1.23E-22 | 1.059701 | CD3D | 2.12E-16 | 0.217173 |
| RPL35 | 7.03E-11 | 0.279359 | LYAR | 3.36E-30 | 1.052247 | RHBDD2 | 3.93E-16 | 0.600858 |
| GADD45B | 8.48E-11 | 0.573909 | ITPRIP | 5.25E-12 | 1.032418 | PHLDA1 | 5.93E-16 | 0.607761 |
| IFITM2 | 1.09E-10 | 0.49524 | GABARAPL1 | 3.04E-36 | 1.00055 | GABARAP | 1.16E-15 | 0.259501 |
| TGFB1 | 1.41E-10 | 0.828257 | BCL2L11 | 5.97E-14 | 0.9962 | ISCU | 1.17E-15 | 0.438631 |
| PPP1R14B | 1.45E-10 | 2.062316 | MAPK1 | 2.32E-10 | 0.99396 | RNASET2 | 1.52E-15 | 0.548621 |
| CD7 | 2.78E-10 | 0.536371 | AGO2 | 8.49E-07 | 0.981801 | ATP6V0E1 | 1.64E-15 | 0.324375 |
| MAFF | 3.54E-10 | 1.181873 | RIN3 | 1.41E-15 | 0.946468 | NDUFA4 | 2.82E-15 | 0.249144 |
| LDHA | 5.47E-10 | 0.491119 | GFOD1 | 4.82E-06 | 0.922757 | TBC1D4 | 7.81E-15 | 1.56279 |
| FLNA | 1.13E-09 | 0.619532 | RBM38 | 2.63E-08 | 0.910542 | UCP2 | 2.73E-14 | 0.345786 |
| SCML4 | 1.20E-09 | 0.809326 | PITPNC1 | 2.45E-15 | 0.904506 | NDFIP1 | 3.86E-14 | 0.4407 |

| | | | | | | | | |
|---|---|---|---|---|---|---|---|---|
| RPL26 | 3.51E-09 | 0.172551 | LRMP | 3.49E-07 | 0.902999 | PPP1R2 | 5.72E-14 | 0.32851 |
| RPLP2 | 4.07E-09 | 0.206126 | FYN | 1.79E-39 | 0.891762 | CMTM7 | 6.47E-14 | 0.665924 |
| CCL4L2 | 5.61E-09 | 0.865443 | SLA2 | 1.39E-27 | 0.886548 | GPSM3 | 7.56E-14 | 0.276982 |
| SAMD3 | 1.24E-08 | 0.716863 | GZMH | 1.09E-32 | 0.877032 | TNFRSF25 | 1.37E-13 | 1.495584 |
| IER2 | 1.24E-08 | 0.492577 | ITGA1 | 1.13E-07 | 0.871298 | TRBV19 | 1.52E-13 | 4.156458 |
| SATB1 | 1.53E-08 | 1.463879 | NR4A3 | 2.61E-09 | 0.867305 | PRDX1 | 1.63E-13 | 0.374501 |
| AOAH | 1.83E-08 | 1.029034 | CCL4 | 4.26E-35 | 0.865559 | M6PR | 1.68E-13 | 0.55105 |
| RPL21 | 2.23E-08 | 0.248196 | RUNX3 | 4.63E-30 | 0.852541 | PGAM1 | 2.16E-13 | 0.3011 |
| RPL3 | 4.01E-08 | 0.200847 | VPS37B | 1.86E-27 | 0.84489 | THEMIS | 2.81E-13 | 0.719678 |
| MAPK1 | 6.16E-08 | 1.237451 | SLAMF7 | 2.90E-07 | 0.841258 | CIRBP | 2.83E-13 | 0.242917 |
| IRF7 | 6.18E-08 | 0.746288 | GLUL | 1.25E-18 | 0.831238 | CXCL13 | 3.40E-13 | 1.319922 |
| CEBPD | 6.19E-08 | 2.311149 | G6PD | 2.03E-10 | 0.809316 | PHTF2 | 9.92E-13 | 0.899101 |
| RPS14 | 7.42E-08 | 0.191345 | HOPX | 2.81E-30 | 0.807676 | GMFG | 1.29E-12 | 0.27434 |
| EIF4A1 | 8.24E-08 | 0.654039 | RPS10 | 3.27E-161 | 0.803774 | YWHAB | 1.41E-12 | 0.21848 |
| IL18RAP | 8.99E-08 | 2.230473 | AC004687.1 | 1.63E-07 | 0.789176 | RPS28 | 1.71E-12 | 0.144399 |
| STOM | 9.75E-08 | 0.610071 | STARD3NL | 8.55E-14 | 0.786588 | SNX9 | 2.96E-12 | 0.921244 |
| PRF1 | 1.06E-07 | 0.590522 | MBP | 2.19E-24 | 0.78364 | FTH1 | 6.06E-12 | 0.235514 |
| GLUL | 1.06E-07 | 0.824716 | MVD | 2.00E-06 | 0.78019 | TRAV8-2 | 7.15E-12 | 4.721155 |
| PAXX | 1.15E-07 | 0.411769 | PIK3R1 | 8.80E-34 | 0.778017 | LINC01943 | 1.22E-11 | 1.174219 |
| CD99 | 1.16E-07 | 0.341354 | RASGEF1B | 8.88E-08 | 0.777567 | ITM2A | 1.59E-11 | 0.365135 |
| TBX21 | 1.25E-07 | 0.752075 | ITM2C | 8.20E-36 | 0.774152 | HSPE1 | 2.29E-11 | 0.324597 |
| CTSA | 1.85E-07 | 0.679958 | PPP2R2B | 2.00E-06 | 0.772637 | TNFAIP8 | 3.29E-11 | 0.51894 |
| RPS18 | 1.87E-07 | 0.183493 | TRBC1 | 1.69E-36 | 0.766934 | RPS26 | 3.42E-11 | 0.229132 |
| MAP3K8 | 2.31E-07 | 0.622651 | CD63 | 4.87E-32 | 0.764707 | ATP6V0E2 | 7.09E-11 | 0.713818 |

| | | | | | | | | |
|---|---|---|---|---|---|---|---|---|
| RPS9 | 2.66E-07 | 0.21065 | ATP8A1 | 2.70E-10 | 0.761868 | HSPH1 | 2.44E-10 | 0.359106 |
| STARD3NL | 2.70E-07 | 0.86525 | PECAM1 | 1.12E-06 | 0.747916 | FBLN7 | 3.12E-10 | 4.026181 |
| TPT1 | 2.97E-07 | 0.17362 | SRRT | 1.93E-18 | 0.744661 | CNBP | 5.05E-10 | 0.191393 |
| GLIPR2 | 4.27E-07 | 0.614833 | KLRG1 | 2.28E-11 | 0.742574 | RILPL2 | 6.23E-10 | 0.527856 |
| RPS7 | 4.28E-07 | 0.168096 | FOSL2 | 4.84E-08 | 0.740611 | COX8A | 7.45E-10 | 0.211056 |
| APOBEC3G | 6.90E-07 | 0.450942 | ADGRE5 | 6.23E-38 | 0.737356 | LBH | 9.31E-10 | 0.327279 |
| TNFSF14 | 7.23E-07 | 0.741522 | NLRC5 | 7.58E-11 | 0.731373 | HERPUD1 | 1.10E-09 | 0.317714 |
| CD55 | 8.34E-07 | 0.726839 | PRKX | 5.65E-08 | 0.728555 | ARPC1B | 1.19E-09 | 0.208696 |
| CD96 | 8.72E-07 | 0.431934 | PARP8 | 1.81E-31 | 0.728483 | NCF4 | 1.32E-09 | 1.644992 |
| SNHG9 | 8.94E-07 | 0.692379 | TBX21 | 1.29E-14 | 0.724098 | JUN | 1.33E-09 | 0.296362 |
| RPLP1 | 1.43E-06 | 0.138775 | RPL36A | 3.79E-130 | 0.719214 | CAPZB | 1.39E-09 | 0.199663 |
| TRGV7 | 1.48E-06 | 1.936313 | TGFB1 | 3.28E-19 | 0.71505 | GNA15 | 1.39E-09 | 1.85859 |
| PLEK | 1.71E-06 | 1.016381 | RAB37 | 4.87E-06 | 0.714814 | SELENOW | 1.42E-09 | 0.251928 |
| GNG2 | 1.71E-06 | 0.433983 | DUSP5 | 3.77E-07 | 0.703539 | MGAT4A | 1.44E-09 | 0.79179 |
| OASL | 2.07E-06 | 0.561558 | CCDC69 | 5.73E-12 | 0.703309 | OST4 | 1.51E-09 | 0.163968 |
| GPR65 | 2.41E-06 | 0.716342 | EIF4A1 | 3.59E-21 | 0.702927 | SERPINH1 | 1.74E-09 | 0.618089 |
| GYG1 | 2.44E-06 | 0.655628 | SEMA4D | 2.63E-21 | 0.700381 | GALM | 1.79E-09 | 0.53102 |
| RPL27A | 2.70E-06 | 0.3069 | NUCB2 | 1.16E-12 | 0.697308 | DNPH1 | 2.01E-09 | 0.589622 |
| RPS27A | 3.03E-06 | 0.159304 | PPP1R16B | 3.06E-09 | 0.693767 | ITGB1 | 2.24E-09 | 0.38594 |
| RPS24 | 3.07E-06 | 0.135112 | USP11 | 1.58E-07 | 0.693714 | TIMP1 | 2.43E-09 | 1.073875 |
| RPL10A | 3.86E-06 | 0.235972 | CCL4L2 | 1.78E-12 | 0.691616 | CD6 | 2.43E-09 | 0.434278 |
| RUNX3 | 3.86E-06 | 0.564319 | GNPTAB | 2.10E-08 | 0.685134 | HSPD1 | 2.43E-09 | 0.32937 |
| CAST | 4.65E-06 | 0.510297 | CLIC3 | 2.56E-09 | 0.684883 | NDUFV2 | 2.99E-09 | 0.389016 |
| C12orf75 | 4.69E-06 | 0.518355 | SLC7A5 | 3.48E-18 | 0.682517 | CD200R1 | 3.25E-09 | 0.951177 |

| | | | | | | | | |
|---|---|---|---|---|---|---|---|---|
| C1orf21 | 5.36E-06 | 1.686926 | SCML4 | 1.34E-14 | 0.681115 | SLAMF1 | 3.45E-09 | 1.145394 |
| PIM1 | 5.36E-06 | 0.495206 | CD7 | 8.29E-44 | 0.665972 | ZFAND2A | 3.55E-09 | 0.496948 |
| NFKB1 | 5.50E-06 | 0.739925 | PRMT9 | 6.08E-07 | 0.664535 | FOXP3 | 4.17E-09 | 5.280272 |
| BCL2A1 | 6.20E-06 | 1.037284 | BIRC2 | 6.60E-11 | 0.664194 | CARD16 | 4.20E-09 | 0.367374 |
| RPL13 | 6.61E-06 | 0.128462 | PTPN22 | 8.29E-19 | 0.660171 | IL6ST | 5.23E-09 | 0.548971 |
| CST7 | 6.61E-06 | 0.518597 | IL21R | 8.84E-11 | 0.659458 | DUSP1 | 5.45E-09 | 0.315107 |
| IGFBP2 | 7.10E-06 | 2.808054 | REL | 1.77E-23 | 0.646033 | PELI1 | 5.77E-09 | 0.737662 |
| ERN1 | 7.22E-06 | 0.672647 | CHST12 | 6.15E-15 | 0.645526 | ITGA4 | 6.15E-09 | 0.359212 |
| KDM6B | 7.43E-06 | 0.927049 | PRF1 | 1.91E-19 | 0.638294 | GK | 6.81E-09 | 1.47895 |
| AC006369.1 | 8.02E-06 | 1.315919 | TRAC | 3.92E-21 | 0.628587 | CUTA | 8.59E-09 | 0.263346 |
| AHNAK | 8.06E-06 | 0.454629 | RPL41 | 1.29E-303 | 0.626936 | ADAM19 | 1.11E-08 | 1.588262 |
| TTC39C | 8.74E-06 | 0.48753 | PRKACB | 3.23E-14 | 0.62334 | PCED1B | 1.15E-08 | 0.542268 |
| PLP2 | 1.01E-05 | 0.3887 | PPP2R5C | 1.48E-16 | 0.622711 | RPL11 | 1.33E-08 | 0.124456 |
| RPLP0 | 1.09E-05 | 0.162442 | CEMIP2 | 1.24E-17 | 0.619114 | CCR7 | 1.39E-08 | 1.306519 |
| RPS4X | 1.32E-05 | 0.159595 | HERC1 | 1.28E-05 | 0.618181 | TRBV27 | 1.45E-08 | 3.482451 |
| RPL27 | 1.36E-05 | 0.240474 | IL2RB | 8.55E-20 | 0.613627 | PKM | 1.83E-08 | 0.256658 |
| ISG15 | 1.46E-05 | 0.537522 | LPIN1 | 5.01E-06 | 0.608843 | GPR183 | 3.24E-08 | 1.264914 |
| RPL7A | 1.52E-05 | 0.155896 | GSTP1 | 6.57E-28 | 0.595596 | TXN | 3.71E-08 | 0.284058 |
| NFKBID | 1.58E-05 | 1.202434 | OGT | 1.14E-06 | 0.594381 | SELENOM | 4.12E-08 | 0.628859 |
| SLAMF7 | 1.62E-05 | 1.017139 | ADAM8 | 1.82E-07 | 0.592217 | DAZAP2 | 4.26E-08 | 0.154075 |
| SYNE1 | 2.08E-05 | 0.669803 | SKIL | 1.32E-07 | 0.588922 | SERF2 | 4.95E-08 | 0.112573 |
| BIN2 | 2.22E-05 | 0.522057 | FAM173A | 1.21E-07 | 0.586562 | UBL5 | 4.95E-08 | 0.163174 |
| PCBP1 | 2.59E-05 | 0.319863 | BLOC1S1 | 5.94E-10 | 0.579245 | CIB1 | 6.15E-08 | 0.218872 |
| PRKACB | 2.75E-05 | 0.569646 | RC3H1 | 8.88E-08 | 0.578359 | DNAJA1 | 6.47E-08 | 0.268042 |

| | | | | | | | | |
|---|---|---|---|---|---|---|---|---|
| RPL39 | 2.87E-05 | 0.136333 | SNHG9 | 1.17E-09 | 0.570271 | SARAF | 7.00E-08 | 0.169493 |
| TC2N | 2.93E-05 | 0.533244 | C12orf75 | 1.79E-16 | 0.569066 | FTL | 8.25E-08 | 0.182103 |
| RPL32 | 3.13E-05 | 0.107032 | LDLRAD4 | 8.78E-06 | 0.565474 | CD37 | 8.88E-08 | 0.162004 |
| LCP1 | 3.14E-05 | 0.308271 | ISG20 | 1.57E-24 | 0.565389 | TESPA1 | 1.11E-07 | 0.737631 |
| PARP8 | 3.34E-05 | 0.447972 | NR4A2 | 9.62E-18 | 0.565109 | UGP2 | 1.35E-07 | 0.477374 |
| PIK3AP1 | 3.85E-05 | 1.134662 | CDK17 | 2.68E-06 | 0.560452 | TSHZ2 | 1.39E-07 | 1.832476 |
| ID2 | 4.09E-05 | 0.491933 | MAZ | 7.79E-07 | 0.56013 | PPP1R15A | 1.71E-07 | 0.244737 |
| PRNP | 4.09E-05 | 0.574766 | STOM | 2.21E-13 | 0.559894 | SH3BGRL | 1.91E-07 | 0.272981 |
| PIK3R1 | 4.18E-05 | 0.433609 | FAM177A1 | 1.08E-16 | 0.559619 | PIM2 | 2.51E-07 | 0.552398 |
| IFITM3 | 5.03E-05 | 1.490239 | CST7 | 8.34E-24 | 0.554816 | SNAP47 | 3.43E-07 | 0.633495 |
| IFNGR1 | 5.39E-05 | 0.78248 | EIF3J | 9.97E-09 | 0.5527 | EEF1G | 3.59E-07 | 0.16011 |
| FASLG | 5.40E-05 | 0.757462 | ZNF331 | 1.48E-16 | 0.546126 | TLK1 | 4.60E-07 | 0.63696 |
| CAMK4 | 6.66E-05 | 0.549061 | STMN1 | 8.04E-08 | 0.545768 | RNASEK | 5.47E-07 | 0.175957 |
| MACF1 | 7.05E-05 | 0.516477 | PLA2G16 | 3.60E-10 | 0.544889 | PFN1 | 7.12E-07 | 0.136326 |
| CD44 | 7.86E-05 | 0.342373 | BRD2 | 1.14E-20 | 0.540548 | DEDD2 | 7.35E-07 | 0.415938 |
| TBCD | 0.000127 | 0.898011 | GLIPR2 | 3.71E-13 | 0.539164 | CDKN1B | 9.35E-07 | 0.339236 |
| ZC3H12A | 0.000135 | 0.556471 | CNOT6L | 1.75E-08 | 0.538048 | APRT | 9.47E-07 | 0.19566 |
| SLC7A5 | 0.000198 | 0.535854 | NFKB1 | 2.59E-08 | 0.536415 | IL32 | 9.56E-07 | 0.17577 |
| EGR1 | 0.000224 | 0.853373 | LY9 | 1.16E-05 | 0.535795 | SPCS1 | 1.10E-06 | 0.214764 |
| PDCD4 | 0.000234 | 0.397259 | CTSA | 9.95E-09 | 0.534703 | ATP5MC2 | 1.27E-06 | 0.15287 |
| IER3 | 0.000246 | 0.834034 | AKAP13 | 1.02E-16 | 0.532693 | UQCR11 | 2.21E-06 | 0.150983 |
| GZMA | 0.000289 | 0.445298 | RANBP2 | 2.48E-09 | 0.520661 | SNX3 | 2.28E-06 | 0.24179 |
| ISG20 | 0.000301 | 0.382359 | MAP3K8 | 1.72E-10 | 0.519691 | TRAV4 | 2.86E-06 | 4.736338 |
| NFE2L2 | 0.000327 | 0.528528 | SLC38A2 | 1.60E-13 | 0.519457 | TRAV27 | 3.41E-06 | 4.490881 |

| | | | | | | | | |
|---|---|---|---|---|---|---|---|---|
| TNF | 0.000332 | 0.52249 | MT2A | 1.18E-17 | 0.518438 | SKP1 | 3.42E-06 | 0.157062 |
| PRMT2 | 0.000347 | 0.416781 | ACTN4 | 8.48E-12 | 0.513333 | RAP1A | 3.62E-06 | 0.311845 |
| CLEC2B | 0.000351 | 0.354415 | LINC01871 | 4.32E-07 | 0.51038 | DOK2 | 4.09E-06 | 0.262599 |
| SH3BGRL3 | 0.000351 | 0.229268 | CLEC2B | 1.11E-20 | 0.509488 | TNFRSF14 | 4.66E-06 | 0.318079 |
| RORA | 0.000451 | 0.56507 | INSIG1 | 7.24E-08 | 0.506346 | COX7C | 4.75E-06 | 0.114029 |
| PTMS | 0.000456 | 0.446933 | PTPRA | 1.28E-06 | 0.504428 | FAS | 4.75E-06 | 0.437967 |
| ABCB1 | 0.000459 | 1.173953 | ZFP36 | 3.10E-18 | 0.502305 | LIMD2 | 5.18E-06 | 0.189687 |
| CD300A | 0.000492 | 1.417403 | IDI1 | 1.41E-13 | 0.497109 | SH3KBP1 | 5.26E-06 | 0.298137 |
| CTSD | 0.000506 | 0.553134 | LASP1 | 1.51E-06 | 0.487588 | TRBV20-1 | 5.30E-06 | 3.124117 |
| PBX4 | 0.000584 | 0.555235 | AC116366.3 | 1.02E-07 | 0.482736 | FAAH2 | 5.41E-06 | 1.729235 |
| AC007384.1 | 0.00059 | 0.861484 | PSMA2 | 5.39E-07 | 0.479795 | KRTCAP2 | 5.59E-06 | 0.18731 |
| CKLF | 0.00062 | 0.359765 | OASL | 3.81E-09 | 0.478269 | CD59 | 6.23E-06 | 0.991598 |
| RPS5 | 0.000639 | 0.182327 | ZFP36L2 | 2.34E-26 | 0.477421 | EID1 | 6.35E-06 | 0.245789 |
| YBX3 | 0.000687 | 1.235966 | AP2B1 | 8.40E-06 | 0.474951 | COMMD6 | 7.57E-06 | 0.128046 |
| KTN1 | 0.000723 | 0.406056 | HCST | 3.27E-39 | 0.4692 | TMEM258 | 7.93E-06 | 0.192632 |
| RPL38 | 0.000769 | 0.198925 | CTSD | 6.74E-06 | 0.467426 | TOX | 8.46E-06 | 0.481873 |
| TRBC2 | 0.000769 | 0.33164 | NFE2L2 | 1.59E-08 | 0.467104 | CCT4 | 8.64E-06 | 0.23568 |
| ITGA5 | 0.000769 | 1.207945 | ATP1B3 | 2.19E-06 | 0.460039 | UQCR10 | 9.20E-06 | 0.190473 |
| RALGAPA1 | 0.000792 | 0.534051 | CD38 | 1.56E-05 | 0.459412 | BCAP31 | 9.86E-06 | 0.264682 |
| AES | 0.000832 | 0.347216 | PDCD4 | 3.54E-12 | 0.458659 | GSTK1 | 1.02E-05 | 0.167127 |
| ZYX | 0.000909 | 0.363744 | PTMS | 2.16E-10 | 0.455262 | CDKN2C | 1.06E-05 | 0.60015 |
| NFKBIZ | 0.000909 | 0.452999 | RASSF1 | 2.79E-06 | 0.453577 | SELL | 1.10E-05 | 1.17707 |
| PERP | 0.000948 | 0.804922 | AKNA | 3.26E-07 | 0.453504 | AHSA1 | 1.39E-05 | 0.345999 |
| STAT4 | 0.001052 | 0.487538 | HERPUD2 | 3.34E-08 | 0.448749 | RNF167 | 1.44E-05 | 0.303012 |

| | | | | | | | | |
|---|---|---|---|---|---|---|---|---|
| SRSF2 | 0.001188 | 0.318747 | JUND | 9.71E-10 | 0.447441 | NPM1 | 1.51E-05 | 0.132602 |
| TAGLN2 | 0.001193 | 0.288102 | TUBA4A | 8.27E-15 | 0.446473 | CCNI | 2.02E-05 | 0.121455 |
| NCF1 | 0.001205 | 0.517122 | TPST2 | 3.00E-06 | 0.445374 | BRK1 | 2.21E-05 | 0.20467 |
| RTKN2 | 0.001238 | 1.60402 | LITAF | 3.96E-14 | 0.445201 | TNFRSF18 | 2.80E-05 | 0.92097 |
| IFNG | 0.001428 | 0.582633 | HLA-DQA1 | 9.16E-10 | 0.443167 | RCAN3 | 3.21E-05 | 0.516221 |
| LRRFIP1 | 0.001492 | 0.331139 | UBAC2 | 1.42E-06 | 0.439441 | TMEM59 | 3.38E-05 | 0.204514 |
| ICAM1 | 0.001607 | 0.862038 | IFITM2 | 8.83E-22 | 0.437588 | ANKRD12 | 3.64E-05 | 0.272256 |
| RPL13A | 0.001628 | 0.303922 | CCSER2 | 2.10E-08 | 0.434074 | NR3C1 | 3.68E-05 | 0.374466 |
| RRAS2 | 0.001649 | 1.154945 | SYAP1 | 4.20E-07 | 0.433628 | SH3BGRL3 | 3.78E-05 | 0.100425 |
| MRPS6 | 0.001658 | 0.40532 | RNF213 | 8.03E-13 | 0.433505 | RPL18 | 4.01E-05 | 0.136292 |
| RPSA | 0.001707 | 0.18466 | RPS29 | 1.90E-138 | 0.432963 | ICAM3 | 4.11E-05 | 0.200815 |
| GRAP2 | 0.001732 | 0.772956 | ARHGAP9 | 2.62E-11 | 0.426283 | TANK | 4.58E-05 | 0.360783 |
| S100A6 | 0.001734 | 0.234329 | OFD1 | 2.57E-05 | 0.421625 | COX5B | 4.75E-05 | 0.13785 |
| RBMS1 | 0.001739 | 0.475881 | TLN1 | 1.86E-05 | 0.419678 | TMA7 | 5.40E-05 | 0.112041 |
| HLA-C | 0.001753 | 0.205217 | DUSP2 | 4.56E-15 | 0.41828 | PTGES3 | 6.58E-05 | 0.163608 |
| UBE2S | 0.00182 | 0.722474 | ETS1 | 4.39E-08 | 0.414094 | CSF1 | 7.83E-05 | 0.473529 |
| ADGRE5 | 0.001842 | 0.354337 | ARF6 | 7.49E-11 | 0.413732 | MAT2B | 8.06E-05 | 0.308792 |
| RPL31 | 0.001986 | 0.251037 | TRBC2 | 6.01E-14 | 0.411397 | BAX | 9.05E-05 | 0.2602 |
| MCOLN2 | 0.002098 | 0.874562 | RSRP1 | 1.72E-10 | 0.408857 | NME3 | 9.37E-05 | 0.345345 |
| ATP1A1 | 0.00214 | 0.36995 | IFITM1 | 4.34E-24 | 0.408819 | BLVRA | 9.65E-05 | 0.843187 |
| GLRX | 0.002216 | 0.479501 | H2AFX | 1.14E-08 | 0.408471 | RPL36AL | 9.75E-05 | 0.101055 |
| EFHD2 | 0.002254 | 0.702922 | MACF1 | 7.13E-06 | 0.406246 | CTSC | 0.0001 | 0.244853 |
| RPS6 | 0.002399 | 0.149773 | SAMD3 | 6.29E-06 | 0.402596 | MAF | 0.00012 | 0.728718 |
| RPL37 | 0.002501 | 0.11925 | FOSB | 8.51E-10 | 0.400418 | TRAV13-1 | 0.00013 | 5.11296 |

| | | | | | | | | |
|---|---|---|---|---|---|---|---|---|
| ANXA2R | 0.002501 | 0.565923 | CCL3 | 1.17E-06 | 0.399983 | ABHD14B | 0.000135 | 0.326256 |
| CCDC85B | 0.002501 | 0.306157 | FLNA | 3.11E-07 | 0.399116 | NDUFC1 | 0.000143 | 0.355428 |
| RPS16 | 0.002519 | 0.175695 | FMNL1 | 1.98E-05 | 0.399061 | TRAV17 | 0.000158 | 3.838664 |
| FLOT1 | 0.00269 | 0.695821 | CEBPZ | 1.18E-05 | 0.397907 | TMEM35B | 0.000171 | 0.479862 |
| MT1X | 0.002709 | 0.424766 | WIPF1 | 2.41E-13 | 0.391602 | ELMO1 | 0.000174 | 0.504413 |
| EIF5B | 0.002724 | 0.500209 | ISG15 | 1.14E-08 | 0.38802 | METTL8 | 0.000175 | 1.285624 |
| ZEB2 | 0.002939 | 0.666705 | FNBP1 | 1.07E-09 | 0.387972 | HMOX1 | 0.000185 | 0.793941 |
| SLA2 | 0.002949 | 0.444839 | KPNA2 | 1.64E-06 | 0.386937 | SOD2 | 0.000206 | 0.402118 |
| SKIL | 0.003033 | 0.572421 | CD96 | 5.69E-12 | 0.380352 | DGKA | 0.000208 | 0.518372 |
| AAK1 | 0.003124 | 0.359077 | EVL | 9.62E-21 | 0.377498 | LTA | 0.000225 | 0.790429 |
| SERTAD1 | 0.003436 | 0.358092 | NEAT1 | 8.21E-07 | 0.376712 | C16orf87 | 0.000229 | 0.703589 |
| KLRC3 | 0.003481 | 1.018827 | IQGAP1 | 1.17E-08 | 0.375593 | CHCHD2 | 0.00028 | 0.101815 |
| TRGC2 | 0.003553 | 1.257511 | RBPJ | 7.90E-06 | 0.374108 | NFKBIA | 0.000296 | 0.201192 |
| ARAP2 | 0.003595 | 0.422931 | IFRD1 | 2.71E-07 | 0.373197 | FXYD2 | 0.000299 | 1.190036 |
| RPL28 | 0.003887 | 0.075065 | CBLB | 4.72E-07 | 0.372876 | SUSD3 | 0.000311 | 0.53837 |
| IL32 | 0.003899 | 0.226923 | NDUFA11 | 1.60E-06 | 0.37231 | HSPA6 | 0.000351 | 0.319307 |
| FEZ1 | 0.004097 | 3.486758 | APOBEC3G | 1.31E-10 | 0.369137 | PRDX2 | 0.000374 | 0.28173 |
| SYNGR1 | 0.004236 | 2.414158 | PHF20 | 1.84E-06 | 0.36875 | ADI1 | 0.000374 | 0.827794 |
| SLC38A2 | 0.004272 | 0.356956 | FDFT1 | 6.48E-06 | 0.368522 | BTG3 | 0.000415 | 0.26538 |
| PPP2R5C | 0.004432 | 0.396747 | GUK1 | 2.05E-21 | 0.36738 | TRBV5-1 | 0.000422 | 4.030981 |
| ARHGEF2 | 0.004709 | 0.885878 | MCL1 | 1.18E-14 | 0.366884 | FOXP1 | 0.000484 | 0.500654 |
| AC004687.1 | 0.004902 | 0.73978 | DDX3X | 7.99E-06 | 0.364472 | RGS2 | 0.000507 | 0.297859 |
| LMO4 | 0.005181 | 0.588692 | TRABD | 2.63E-05 | 0.36408 | TRADD | 0.000529 | 0.511786 |
| PILRB | 0.005444 | 1.079263 | MAPRE2 | 8.00E-08 | 0.354967 | PARK7 | 0.000538 | 0.138909 |

| | | | | | | | | |
|---|---|---|---|---|---|---|---|---|
| HMGA1 | 0.005444 | 0.518266 | MT-ATP8 | 7.17E-21 | 0.350902 | EIF1 | 0.000567 | 0.108592 |
| RNF213 | 0.005936 | 0.288867 | SP100 | 1.46E-08 | 0.349383 | S100A6 | 0.000567 | 0.108639 |
| CDC42EP3 | 0.006135 | 0.33132 | METTL9 | 1.81E-05 | 0.349005 | SERINC5 | 0.000567 | 0.902142 |
| FYN | 0.006831 | 0.354529 | MYADM | 4.86E-07 | 0.34537 | PHPT1 | 0.000579 | 0.314058 |
| RPS17 | 0.00699 | 0.321951 | YBX1 | 2.03E-24 | 0.344799 | FLT3LG | 0.000579 | 0.360586 |
| TPM4 | 0.007247 | 0.363838 | HLA-DPB1 | 2.08E-08 | 0.342961 | DBNDD2 | 0.000579 | 1.172023 |
| FOSL2 | 0.007265 | 0.699495 | MT-CO3 | 7.33E-17 | 0.341275 | MIF | 0.000584 | 0.094341 |
| IQGAP1 | 0.007411 | 0.306631 | STARD7 | 8.03E-06 | 0.338312 | FKBP4 | 0.000621 | 0.294548 |
| SERPINB9 | 0.00782 | 0.514635 | SRSF2 | 2.91E-09 | 0.33397 | TRAV8-6 | 0.000623 | 3.63196 |
| IKZF3 | 0.007914 | 0.441678 | PRDX6 | 8.67E-08 | 0.332553 | EEF1A1 | 0.000644 | 0.132825 |
| DDAH2 | 0.00808 | 0.666506 | PRRC2C | 3.04E-07 | 0.328447 | CD79B | 0.000649 | 1.429338 |
| DYNC1H1 | 0.008229 | 0.420957 | SFPQ | 3.96E-07 | 0.326295 | RAN | 0.000666 | 0.131368 |
| SLC10A3 | 0.008932 | 0.587656 | LYST | 4.79E-06 | 0.322523 | CALM3 | 0.00067 | 0.15142 |
| SUPT3H | 0.008968 | 0.547019 | MT-ND4 | 4.09E-13 | 0.3193 | IL10RA | 0.000686 | 0.205106 |
| ARHGAP15 | 0.008988 | 0.375745 | CCNH | 1.47E-05 | 0.319015 | THEM4 | 0.000701 | 0.84835 |
| NR4A3 | 0.009402 | 0.739231 | IER2 | 3.20E-09 | 0.318867 | CCDC141 | 0.000741 | 0.974759 |
| ANXA2 | 0.009599 | 0.363058 | AMD1 | 2.63E-05 | 0.317007 | EDF1 | 0.000754 | 0.1024 |
| KLRK1 | 0.009913 | 1.11831 | PRKCH | 2.18E-06 | 0.316425 | NDUFA1 | 0.000756 | 0.106591 |
| RNF166 | 0.009937 | 0.384464 | CXCR4 | 5.58E-08 | 0.310805 | CLEC2D | 0.000756 | 0.270806 |
| AUTS2 | 0.010094 | 1.582313 | FOS | 3.15E-07 | 0.310251 | SUB1 | 0.000789 | 0.138574 |
| TRGV10 | 0.01017 | 0.638187 | HLA-DRB1 | 1.93E-06 | 0.310242 | PSAP | 0.000789 | 0.255098 |
| FAM177A1 | 0.010844 | 0.347924 | TSC22D3 | 2.76E-06 | 0.305899 | PPIB | 0.000814 | 0.093893 |
| MYADM | 0.011568 | 0.350819 | ZYX | 1.30E-06 | 0.304157 | SGK1 | 0.000863 | 0.577014 |
| PITPNC1 | 0.012141 | 0.546625 | RBM39 | 1.37E-08 | 0.302733 | CNST | 0.000871 | 0.641598 |

| | | | | | | | | |
|---|---|---|---|---|---|---|---|---|
| MT1F | 0.012183 | 0.532184 | SUN2 | 9.77E-06 | 0.300509 | LGALS1 | 0.000915 | 0.204266 |
| FKBP2 | 0.012193 | 0.481394 | RPL13A | 7.41E-07 | 0.300488 | ASB2 | 0.000929 | 0.909831 |
| THAP2 | 0.012201 | 0.817448 | GNAS | 6.91E-06 | 0.300219 | KDSR | 0.000993 | 0.859211 |
| CHD4 | 0.012481 | 0.607531 | IRF1 | 1.24E-07 | 0.299588 | COX6B1 | 0.00106 | 0.099818 |
| RPL14 | 0.01252 | 0.111474 | HLA-C | 2.20E-30 | 0.294732 | CCR4 | 0.001092 | 2.125206 |
| IKZF1 | 0.012803 | 0.343693 | MT-CO2 | 1.82E-19 | 0.293547 | LINC01480 | 0.001164 | 0.926654 |
| ABRACL | 0.013197 | 0.299584 | FUS | 4.36E-06 | 0.293045 | EMP3 | 0.001248 | 0.149544 |
| NCOA7 | 0.013506 | 0.58984 | LAG3 | 2.05E-06 | 0.291931 | TSPO | 0.001265 | 0.183197 |
| RPL19 | 0.013835 | 0.090095 | MT-ATP6 | 2.04E-12 | 0.289396 | CHMP2A | 0.00139 | 0.365103 |
| ABHD17A | 0.013893 | 0.664926 | YPEL5 | 6.32E-06 | 0.287443 | SUMO2 | 0.001398 | 0.106117 |
| TOP1 | 0.013893 | 0.369235 | GYPC | 1.18E-07 | 0.287047 | SESN3 | 0.001519 | 0.917242 |
| CASP4 | 0.013893 | 0.352521 | MT-ND3 | 9.82E-11 | 0.281082 | SDF4 | 0.001617 | 0.287708 |
| IRF1 | 0.013893 | 0.280767 | RPL37A | 3.27E-32 | 0.280425 | MRPL18 | 0.001633 | 0.292117 |
| NUCB2 | 0.014727 | 0.4908 | LCP1 | 7.47E-10 | 0.2803 | MAGEH1 | 0.001684 | 0.856466 |
| GBP5 | 0.014994 | 0.283873 | PAXX | 4.87E-06 | 0.279983 | TRBV7-9 | 0.001832 | 3.280874 |
| XBP1 | 0.015048 | 0.314194 | TAGLN2 | 2.42E-09 | 0.276995 | BCAS2 | 0.001905 | 0.241367 |
| SP100 | 0.015262 | 0.284396 | MT-ND5 | 2.61E-09 | 0.276146 | ENTPD1 | 0.001962 | 0.838358 |
| TRGV8 | 0.016265 | 1.37384 | PGK1 | 1.17E-08 | 0.274573 | ATP5PF | 0.001994 | 0.179216 |
| ATP5ME | 0.016361 | 0.238009 | CALM2 | 4.67E-07 | 0.268119 | ABRACL | 0.002082 | 0.179797 |
| BIRC2 | 0.016496 | 0.466144 | MT-CO1 | 1.14E-22 | 0.267925 | INPP4B | 0.002223 | 0.378852 |
| RIPOR2 | 0.017415 | 0.462184 | PTPRC | 8.25E-13 | 0.266437 | CD8B2 | 0.002226 | 4.133559 |
| RELA | 0.017699 | 0.492923 | MT-ND4L | 1.08E-12 | 0.257862 | GRSF1 | 0.002235 | 0.371555 |
| RPL17 | 0.01778 | 0.142215 | TAPBP | 1.17E-05 | 0.247159 | LIMS1 | 0.002247 | 0.410226 |
| IL12RB2 | 0.01785 | 1.448177 | ITGB2 | 3.64E-08 | 0.247085 | CSTB | 0.002267 | 0.155897 |

| | | | | | | | | |
|---|---|---|---|---|---|---|---|---|
| RHOF | 0.018 | 0.292548 | CD99 | 1.46E-09 | 0.243323 | TRIB1 | 0.002383 | 1.027339 |
| HNRNPUL1 | 0.01821 | 0.341933 | LDHA | 1.15E-05 | 0.24021 | GAPDH | 0.002454 | 0.106762 |
| SRRM2 | 0.018286 | 0.385313 | PABPC1 | 9.42E-13 | 0.22286 | SSR2 | 0.002487 | 0.114094 |
| IMPDH1 | 0.018499 | 0.864765 | MT-ND1 | 2.14E-05 | 0.219172 | TRAF3IP3 | 0.00249 | 0.246485 |
| PDE4B | 0.01911 | 0.366771 | RPL21 | 7.03E-19 | 0.218293 | PFDN5 | 0.00252 | 0.06859 |
| ZBTB16 | 0.019185 | 2.70271 | DDX5 | 5.39E-07 | 0.213961 | RORA | 0.002798 | 0.352512 |
| LY6E | 0.019221 | 0.255021 | PSME1 | 3.97E-10 | 0.210032 | EPC1 | 0.00286 | 0.194774 |
| RMRP | 0.019659 | 0.932257 | RPL23A | 1.18E-11 | 0.131811 | CREM | 0.002869 | 0.223949 |
| GIMAP1 | 0.020191 | 0.460671 | RPS2 | 1.17E-06 | 0.077854 | STIP1 | 0.002955 | 0.297642 |
| RPL18A | 0.020544 | 0.082833 | RPS21 | 6.12E-11 | 0.055204 | CCR6 | 0.00298 | 1.621466 |
| TMC6 | 0.020753 | 0.328153 | RPS24 | 1.75E-08 | 0.045525 | TRBV2 | 0.003075 | 3.122037 |
| CPNE7 | 0.020993 | 0.727111 | RPL37 | 6.29E-08 | 0.039619 | EIF3H | 0.003315 | 0.129843 |
| ARL4C | 0.021254 | 0.320774 | RPS27 | 1.34E-12 | 0.033811 | MAL | 0.003373 | 1.245009 |
| RARA | 0.021261 | 0.584862 | TMSB4X | 1.73E-05 | 0.017515 | ENO1 | 0.003447 | 0.13421 |
