# Supplementary material for: Exhausted intratumoral Vδ2− γδ T cells in human kidney cancer retain effector function
Source: Nat Immunol. 2023 Mar 16;24(4):612–24. doi: 10.1038/s41590-023-01448-7 (PMC10063448; doi:10.1038/s41590-023-01448-7)
Supplement: Supplementary file 3 — Custom oligonucleotides used for γδ TCR amplification. [file 41590_2023_1448_MOESM3_ESM.pdf]

Supplementary Table 2. Custom oligonucleotides used for γδ TCR amplification.

Oligo Name	DNA Sequence
TRDC-1R-10x	TCAAAGTCAGTGGAGTGCACAGT
TRDC-2R-10x	CCTTCACCAGACAAGCGACA
TRGC-1R-10x	TGTATGTGTCGTTAGTCTTCATGGTG
TRGC-2R-10x	CAAAGGTATGTTCCAGCCTTCTG
